# Rapid prediction of possible inhibitors for SARS-CoV-2 main protease using docking and FPL simulations†

**DOI:** 10.1039/d0ra06212j

**Published:** 2020-08-28

**Authors:** Minh Quan Pham, Khanh B. Vu, T. Ngoc Han Pham, Le Thi Thuy Huong, Linh Hoang Tran, Nguyen Thanh Tung, Van V. Vu, Trung Hai Nguyen, Son Tung Ngo

**Affiliations:** Graduate University of Science and Technology, Vietnam Academy of Science and Technology Hanoi Vietnam; Institute of Natural Products Chemistry, Vietnam Academy of Science and Technology Hanoi Vietnam; School of Biotechnology, International University Ho Chi Minh City Vietnam; Vietnam National University Ho Chi Minh City Vietnam; Faculty of Pharmacy, Ton Duc Thang University Ho Chi Minh City Vietnam; Faculty of Civil Energeering, Ho Chi Minh University of Technology (HCMUT) Ho Chi Minh Vietnam; Institute of Materials Science, Vietnam Academy of Science and Technology Hanoi Vietnam; NTT Hi-Tech Institute, Nguyen Tat Thanh University Ho Chi Minh City Vietnam; Laboratory of Theoretical and Computational Biophysics, Ton Duc Thang University Ho Chi Minh City Vietnam ngosontung@tdtu.edu.vn; Faculty of Applied Sciences, Ton Duc Thang University Ho Chi Minh City Vietnam

## Abstract

Originating for the first time in Wuhan, China, the outbreak of SARS-CoV-2 has caused a serious global health issue. An effective treatment for SARS-CoV-2 is still unavailable. Therefore, in this study, we have tried to predict a list of potential inhibitors for SARS-CoV-2 main protease (Mpro) using a combination of molecular docking and fast pulling of ligand (FPL) simulations. The approaches were initially validated over a set of eleven available inhibitors. Both Autodock Vina and FPL calculations produced consistent results with the experiments with correlation coefficients of *R*_Dock_ = 0.72 ± 0.14 and *R*_W_ = −0.76 ± 0.10, respectively. The combined approaches were then utilized to predict possible inhibitors that were selected from a ZINC15 sub-database for SARS-CoV-2 Mpro. Twenty compounds were suggested to be able to bind well to SARS-CoV-2 Mpro. Among them, five top-leads are *periandrin V*, *penimocycline*, *cis-p-Coumaroylcorosolic acid*, *glycyrrhizin*, and *uralsaponin B*. The obtained results could probably lead to enhance the COVID-19 therapy.

## Abstract 2

A combination of Autodock Vina and FPL calculations suggested that *periandrin V*, *penimocycline*, *cis-p-Coumaroylcorosolic acid*, *glycyrrhizin*, and *uralsaponin B* are able to bind well to SARS-CoV-2 Mpro.

## Graphical abstract

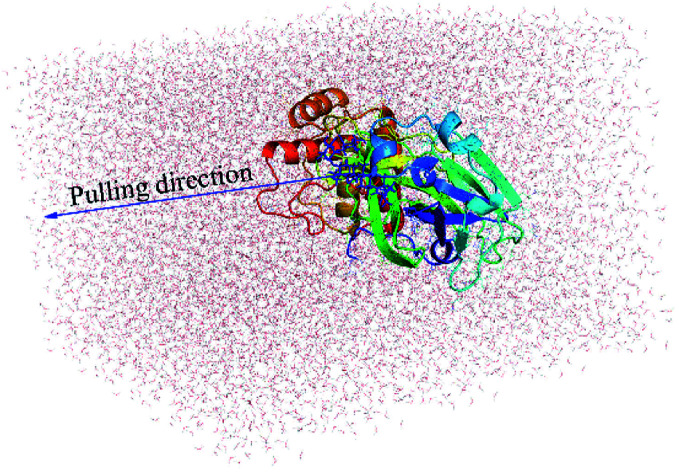

## Introduction

The novel coronavirus, named SARS-CoV-2 or 2019-nCoV, caused the COVID-19 disease, which is an ongoing global pandemic. First cases of COVID-19 infection were reported in Wuhan, Hubei, China in December 2019.^1–3^ The virus was found to be able to transmit from human to human.^4^ It has been suggested that SARS-CoV-2 particularly can transmit through air/aerosols since the virus was found to remain viable and infectious in such environments for more than three hours.^5^ The novel coronavirus causes severe acute respiratory syndromes, which have resulted in hundreds of thousands of deaths worldwide.^6,7^ Moreover, the intermediate host is still undetected, although the original reservoir is indicated as the bat.^8^ Understanding the spread of the virus thus becomes more difficult. The current global health crisis caused by COVID-19 has called for urgent research and development of an efficient antiviral drug.

SARS-CoV-2 and SARS-CoV share about 82% similarity in their RNA genomes. The genomes of Coronaviruses range from 26 to 32 kb in length. The viruses thus have the largest sequence among RNA viruses.^9,10^ There are more than 20 different proteins encoded by the genomes of SARS-CoV-2. In particular, SARS-CoV-2 Mpro is known as one of the most critical viral proteins. It should be noted that SARS-CoV-2 Mpro shares more than 96% similarity to SARS-CoV Mpro. During the viral translation, the SARS-CoV-2 Mpro cleaves eleven polyproteins to polypeptides, which are necessary for the transcription and replication of the virus.^10^ The SARS-CoV-2 Mpro is selected as one of the most potent drug targets for inhibiting viral proliferations.^11,12^ Therefore, numerous studies have been conducted *via* experimental and computational approaches in order to search for potential small-molecule inhibitors that can effectively block the activity of this protease.^11–18^

It should be noted that computer-aided drug design (CADD) can significantly reduce the time and cost of developing a therapy.^19,20^ In the CADD approach, the ligand-binding free energy Δ*G* can be calculated through MD simulations. This metric is linked with the experiment through formula Δ*G*_bind_ = *RT *ln(*k*_i_), where *k*_i_ is the inhibition constant, *T* is the absolute temperature, and *R* is the gas constant. In some cases, IC_50_ is assumed to be equal to *k*_i_ in order to estimate the experimental binding free energy (Δ*G*_EXP_).^15,21,22^ Because the metric reveals the binding mechanism between biomolecules,^19^ an accurate and precise investigation of the ligand-binding free energy is tremendously critical for searching potential inhibitors.^23^ In this study, the potential candidates from ZINC15 in man compounds,^24^ which can bind to the SARS-CoV-2 Mpro, were screened using a combined approach of molecular docking and FPL simulations. It should be noted that this combined computational scheme was previously validated by testing on eleven available inhibitors whose computed binding free energies were in good agreement with respect to experiments.^11–14^ Our present study suggested that twenty compounds were able to bind with high affinity to SARS-CoV-2 Mpro. These compounds can become promising leads for developing drugs against the COVID-19 disease.

## Materials and methods

### Structure of ligands and SARS-CoV-2 Mpro

The crystal structure of monomeric SARS-COV-2 Mpro was obtained from the Protein Data Bank with the identity of 6Y2F.^11^ It should be noted that the computational investigations of promising inhibitors for SARS-CoV-2 Mpro are possible for the monomeric form^25,26^ because the SARS-CoV-2 Mpro dimer interface does not contain the substrate-binding cleft.^11,12^ Ligand structures were taken from the ZINC15 in man only and the PubChem database.^24,27^

### Molecular docking simulations

The ligands were docked to the SARS-CoV-2 Mpro using the Autodock Vina version 1.1 package.^28^ The parameter of the docking approach was preferred according to the previous study^15,29^ in which the parameter of exhaustiveness was set to the default value of 8. The best docking conformations were chosen as having the largest binding affinity. The grid center was designated as the center of mass of compound α-ketoamide 13b.^11^ The docking grid was chosen as 2.6 × 2.6 × 2.6 nm according to the recent work.^15^

### Fast pulling of ligand (FPL) simulations

GROMACS version 5.1.5 (ref. 30) was utilized to simulate the unbinding process of a ligand pulled out of the binding site of the SARS-CoV-2 Mpro. The protein and ions were parameterized *via* the Amber99SB-ILDN force field.^31^ The TIP3P model was utilized for representing water molecules.^32^ The general Amber force field (GAFF)^33^ was used to represent the ligand *via* AmberTools18.^34^ The ACPYPE^35^ protocol was used to transform AMBER to GROMACS formats. In particular, the ligand atomic charges were fitted using the restrained electrostatic potential (RESP) method,^36^ which is based on DFT calculations with the B3LYP functional and 6-31G(d,p) basis set. It should be noted that the combination of the force fields was preferred since it is one of the most solutions for free energy assessment.^37,38^

**Fig. 1:**
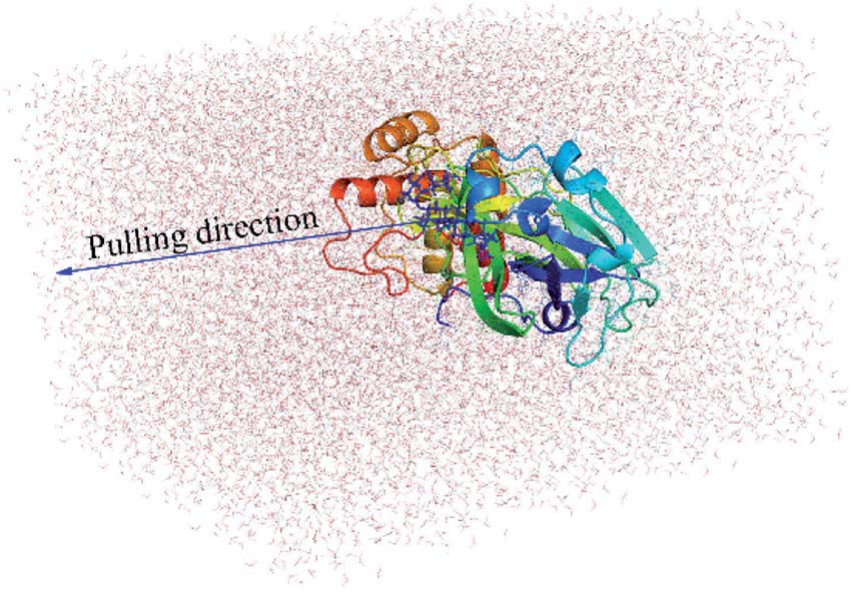
Initial conformation of FPL simulations of the SARS-CoV-2 Mpro + *periandrin V*.

The complex SARS-CoV-2 + ligand was initially introduced into a rectangular PBC (periodic boundary conditions) box with a size of 9.83 × 5.92 × 8.70 nm (Fig. 1), which is similar to the recent study.^15^ The complex system thus consists of more than 50 000 atoms including the SARS-CoV-2 Mpro, inhibitor, water molecules, and counterbalanced ions (Na^+^). The solvated SARS-CoV-2 Mpro + ligand system was first minimized *via* the steepest descent approach. The 0.1 ns of NVT and 2.0 ns of NPT imitations were then followed to relax the complex system, in which the SARS-CoV-2 Mpro *C*_α_ atoms were positionally restrained using a slight harmonic force. Finally, the ligand was forced to move out of the SARS-CoV-2 Mpro binding pocket by applying an external harmonic force at a pulling speed of *k* = 0.005 nm ps^−1^ and a spring constant of *v* = 600 kJ mol^−1^ nm^−2^. The pulling speed and spring constant were chosen to be the same as in the previous works.^15,39,40^ During steered-MD simulations, the ligand displacement and the applied pulling force were recorded every 0.1 ps that would be used to estimate the ligand binding affinity.^39^ Totally, the FPL calculations were independently performed 8 times to guarantee the sufficient sampling.

### Analyzed tools

The ligand protonation state was predicted using the Chemicalize tools (http://www.chemicalize.com), a website application of the ChemAxon. The error of computations was computed through 1000 rounds of the bootstrapping method.^41^ The protein-ligand interaction illustration was prepared *via* the LigPlot++ program.^42^

## Results and discussion

### Molecular docking simulation

**Fig. 2:**
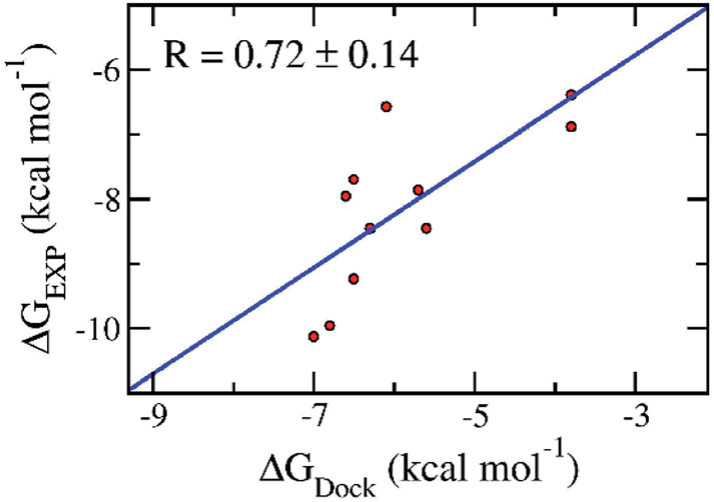
Correlation between molecular docking and experiment. The error of the correlation coefficient was determined *via* 1000 rounds of the bootstrapping method.^41^

Molecular docking simulations are normally employed to probe the binding affinity between ligands and proteins. Autodock Vina,^28^ an open-source docking protocol, is widely used for this purpose. The binding affinity between the ligands and the SARS-CoV-2 Mpro was efficiently estimated using Autodock Vina.^28^ The calculated binding affinity (Table 1) is consistent with the respect to the experimental binding affinity with an estimated correlation coefficient of *R*_Dock_ = 0.72 ± 0.14 (*cf.*Table 1 and Fig. 2).^11–14^ The obtained values are in good agreement with the docking results of these ligands to SARS-CoV-2 Mpro dimer (PDB ID 6XBG)^43^ with a value of *R*^Dimer^_Dock_ = 0.74 (Fig. S1 of the ESI† file). Details are shown in Table S1 and Fig. S1 of the ESI file.† The consistency reveals that the monomer SARS-CoV-2 Mpro can be used as a target for the computer-aided drug design aiming to prevent SARS-CoV-2 Mpro. Moreover, the root-mean-square error (RMSE) with respect to the experiment was estimated as RMSE = 2.42 ± 0.22 kcal mol^−1^ (Fig. 2).^11–14^ It should be noted that the obtained results are consistent with the recent work which reported the corresponding values of *R*_Dock_ = 0.82 ± 0.08 and RMSE = 2.28 ± 0.21 kcal mol^−1^.^44^

**Table 1:** The obtained values of the docking simulations

No.	Name	ΔG_Dock_^a^	ΔG_EXP_^b^
1	11r	−6.5	−9.23
2	13a	−6.5	−7.70
3	13b	−6.3	−8.45
4	11a	−6.8	−9.96
5	11b	−7.0	−10.13
6	*Carmofur*	−5.7	−7.86
7	*Disulfiram*	−3.8	−6.89
8	*Ebselen*	−5.6	−8.45
9	*PX-12*	−3.8	−6.39
10	*Shikonin*	−6.1	−6.58
11	*Tideglusib*	−6.6	−7.95

a The docking affinity was gained using the Autodock Vina package.

b The experimental binding free energy Δ*G*_EXP_ was roughly computed *via* the reported IC_50_ (ref. 11–14) with a supposition that the IC_50_ value is equal to the inhibition constant *k*_i_. The unit is in kcal mol^−1^.

**Fig. 3:**
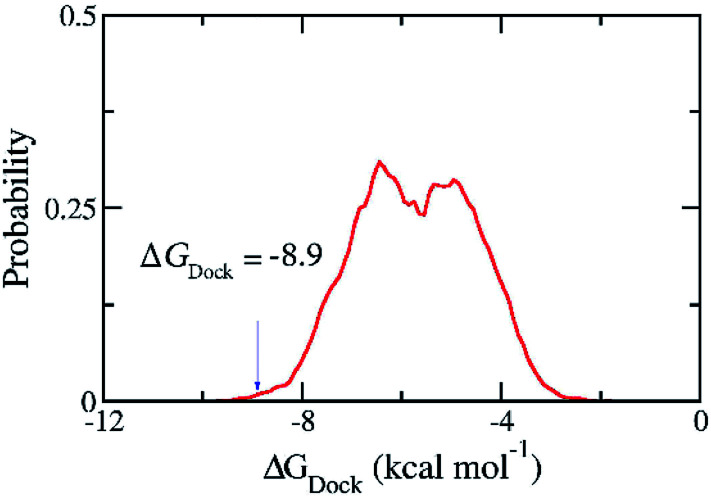
Distribution of the docking energy between 36 089 ZINC15 in man only compounds and the SARS-CoV-2 Mpro. The results were gained using Autodock Vina.

The good docking performance for 11 ligands as shown above gives us the confidence to carry out docking calculations for 36 090 compounds in ZINC15 in man only compounds using the Autodock Vina package.^28^ However, the compound ZINC000169876613 was skipped because it contains the element silicon for which the docking package has no parameters. The estimated binding free energies for 36 089 compounds ranges from −1.8 to −9.9 kcal mol^−1^ and have the median of −5.72 kcal mol^−1^ and the standard deviation of 1.20 kcal mol^−1^. We selected one hundred compounds with binding energy towards the SARS-CoV-2 Mpro lower than −8.9 kcal mol^−1^ (Fig. 3) for further investigations using MD simulations. However, thirty-nine of them were discarded from the set since they were just different in protonation states of the same molecules. Overall, sixty-one compounds with two-dimensional interaction diagrams with SARS-CoV-2 Mpro (Table S2 of the ESI file†) were investigated the ligand-dissociation process using FPL simulations.

### Estimating ligand affinity using FPL simulations

**Fig. 4:**
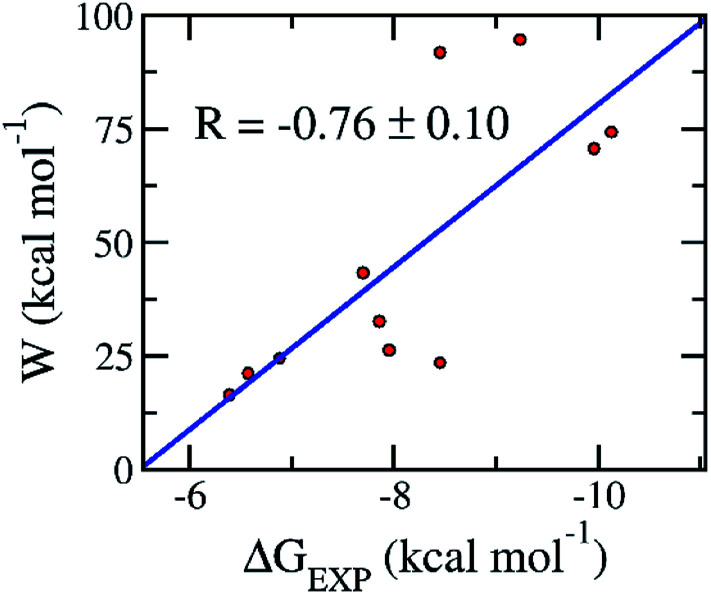
Association between the average of the pulling work *W* and the binding free energy Δ*G*_*EXP*_ of the respective experiments. Computed values were obtained *via* the FPL simulations. Experimental metrics were roughly estimated *via* the reported IC_50_ (ref. 11–14) with a hypothesis that the IC_50_ value is equal to the inhibition constant *k*_*i*_ in the recent publications.^11–14^ The linear regression between pulling work and the experiment is *W* = −17.993 × Δ*G*_EXP_−98.852.

Although, the docking protocol produces appropriate results compared with the experiments (Fig. 2),^11–13^ not considering the receptor dynamics and limiting the number trial position of ligands may cause inaccurate prediction. A more accurate and precise method would be normally employed to refine the docking observation.^22,45^ Moreover, the FPL calculation commonly offers accurate and precise results with a reasonable CPU time consumption.^46^ Furthermore, it should be noted that FPL simulations were successfully used in the previous work^15^ to correctly rank the ligand-binding affinity of the α-ketoamide 11r, 13a, and 13b^11^ to the SARS-CoV-2 Mpro. The validated calculations were also performed over the additionally available inhibitors including 11a, 11b, *carmofur*, *disulfiram*, *ebselen, PX-12*, *shikonin*, and *tideglusib.*^12–14^ The obtained results are revealed in Table 2 and S2 of the ESI file.† In particular, the mean pulling work (*W*) of eleven inhibitors falls in the range from 16.5 ± 1.7 to 94.6 ± 5.0 kcal mol^−1^, giving a median of 47.2 ± 8.6 kcal mol^−1^. Moreover, the average of the rupture forces forms in the range from 321.2 ± 26.5 to −884.2 ± 36.5 pN, giving an average value of 530.2 ± 62.6 pN. The calculated metrics are in good agreement with the respective experiments^11–14^ because the correlation coefficient between the mean pulling work and experimental values is *R*_W_ = −0.76 ± 0.10 (Fig. 4). The calculated error was computed through 1000 rounds of the bootstrapping method.^41^ Furthermore, the sign of the correlation coefficient *R*_W_ implied that the ligand with a stronger binding affinity requires a larger pulling work to dissociate from the SARS-CoV-2 Mpro. Therefore, from linear regression, we could estimate the relation between the binding free energy Δ*G*^Pre^_FPL_ and the pulling work as

1 Δ*G*^Pre^_FPL_ = −0.056 × *W* − 5.512

**Table 2:** The obtained values of the FPL calculations

No.	Name	*F*_Max_ a	*W* b	Δ*G*_EXP_^c^
1	11r	857.5 ± 38.7^d^	94.6 ± 5.0^d^	−9.23
2	13a	496.0 ± 32.5^d^	43.3 ± 3.9^d^	−7.70
3	13b	884.2 ± 36.5^d^	91.9 ± 3.6^d^	−8.45
4	11a	701.3 ± 54.1	70.7 ± 5.9	−9.96
5	11b	718.7 ± 46.8	74.3 ± 4.4	−10.13
6	*Carmofur*	421.5 ± 23.9	32.6 ± 1.8	−7.86
7	*Disulfiram*	371.3 ± 20.3	24.5 ± 1.9	−6.89
8	*Ebselen*	381.0 ± 34.0	23.5 ± 2.5	−8.45
9	*PX-12*	321.3 ± 26.5	16.5 ± 1.7	−6.39
10	*Shikonin*	327.9 ± 24.4	21.2 ± 2.1	−6.58
11	*Tideglusib*	351.8 ± 32.4	26.3 ± 2.4	−7.95

a The obtained value of the mean rupture force *F*_Max_.

b The recorded metric of the pulling work *W*.

c The experimental binding free energy Δ*G*_EXP_ was coarsely estimated *via* the reported IC_50_ (ref. 11–14) with a supposition that the IC_50_ value is equal to the inhibition constant *k*_i_.

d The values were reported in the previous work.^15^ The calculated error was the standard error of the average. The unit is in kcal mol^−1^.

The precision of the FPL estimation was evaluated by the RMSE with linear regression, giving RMSE_W_ = 1.03 ± 0.14 kcal mol^−1^. The small value of RMSE implies that the FPL simulations can discriminate ligands, revealing similar binding free energies. The error was estimated by the standard deviation of 1000 bootstrap samples.^41^ In addition, the measured value *W* is highly correlated (*R* = 0.79) with the number of residues forming SC contacts with the respective ligand (*cf.* Table S1 of the ESI†). The observed agreement implied that the van der Waals interactions probably dominate the binding process of a ligand to SARS-CoV-2 Mpro. It is in good agreement with the results obtained by the other methods.^26,44^ Overall, the FPL calculations are effective protocol to appraise the ligand-binding affinity of the SARS-CoV-2 Mpro with suitable accuracy and precision.

The FPL calculations were thus applied to evaluate the binding free energy of docking-top-lead compounds to the SARS-CoV-2 Mpro, which consists of sixty-one compounds, as listed in Tables 3 and S3 of the ESI file.† The mean rupture forces and mean pulling works were found to diffuse in the range from 389.5 ± 20.9 to 822.4 ± 40.0 pN and 32.9 ± 2.6 to 94.1 ± 4.7 kcal mol^−1^, respectively. Particularly, the median of the corresponding metrics are 574.5 pN and 57.9 kcal mol^−1^, respectively. Moreover, the predicted binding free energies between ligands and the SARS-CoV-2 Mpro were calculated using eqn (1). The value Δ*G*^Pre^_FPL_ was thus obtained and is shown in Tables 3 and S3 of the ESI file.† It may be argued that a ligand with an estimated binding free energy, Δ*G*^Pre^_FPL_, less than −9.00 kcal mol^−1^ may be able to inhibit the activity of the SARS-CoV-2 Mpro, which would adopt the inhibition constant *k*_*i*_ in the sub-micromolar range or smaller.^47^ Therefore, we expect that twenty such compounds will be probable inhibitors for the SARS-CoV-2 Mpro activity (Table 3) because of their strong binding affinity. In addition, we may argue that the other compounds, described in Table S3 of the ESI file,† probably have less effect on the structure of SARS-CoV-2 Mpro.

**Table 3:** The obtained values of the docking and FPL simulations

No.	ZINC ID	Name	Δ*G*_Dock_^a^	*F*_Max_ b	*W* c	Δ*G*^Pre^_FPL_^d^
1	ZINC000256110404	*Periandrin V*	−9.1	782.7 ± 39.0	94.1 ± 4.7	−10.76
2	ZINC000085537131	*Penimocycline*	−9.0	798.3 ± 51.2	92.8 ± 7.0	−10.69
3	ZINC000100783644	*cis-p-Coumaroylcorosolic acid*	−8.9	822.4 ± 40.0	89.5 ± 4.1	−10.51
4	ZINC000253527863	*Glycyrrhizin*	−9.3	598.4 ± 43.2	86.2 ± 8.2	−10.32
5	ZINC000256105139	*Uralsaponin B*	−9.7	690.6 ± 33.7	83.6 ± 3.0	−10.17
6	ZINC000100783815	*3-trans-Caffeoyltormentic acid*	−8.9	731.8 ± 53.0	77.5 ± 4.1	−9.83
7	ZINC000004214527	*Triamcinolone Benetonide*	−8.9	664.0 ± 21.6	74.8 ± 2.3	−9.68
8	ZINC000028642721	*Sennidin A*	−9.5	779.7 ± 58.8	74 ± 5.2	−9.64
9	ZINC000100783890	*23-trans-p-Coumaroyloxytormentic acid*	−9.2	566.7 ± 15.1	72.9 ± 3.4	−9.58
10	ZINC000098052857	*Evans Blue*	−8.9	670.2 ± 56.1	72.7 ± 6.8	−9.56
11	ZINC000100783691	*Sanguisorbin B*	−8.9	616.9 ± 32.9	71.6 ± 3.2	−9.51
12	ZINC000095619992	*Licoricesaponin C2*	−8.9	616.3 ± 44.2	69.9 ± 7.0	−9.41
13	ZINC000118937488	*Withangulatin A*	−9.0	703.5 ± 31.5	68.5 ± 3.7	−9.33
14	ZINC000100783660	*trans-3-Feruloylcorosolic acid*	−9.2	654.9 ± 23.3	67.3 ± 2.5	−9.26
15	ZINC000100777487	*Physalin D*	−8.9	682.3 ± 32.9	65.8 ± 2.8	−9.18
16	ZINC000004879678	*Guamecycline*	−9.6	565.2 ± 24.8	64.7 ± 4.8	−9.12
17	ZINC000150354128	*Bis(4-methoxybenzoyl)-3a,29-dihydroxy-8-multifloren-7-one*	−8.9	564.8 ± 39.4	63.8 ± 3.1	−9.07
18	ZINC000004215464	*Cortisuzol*	−9.2	579.3 ± 38.9	63.5 ± 4.2	−9.05
19	ZINC000100774273	*Rubroskyrin*	−8.9	696.1 ± 51.3	62.9 ± 5.1	−9.02
20	ZINC000073224787	*Tirilazad Mesylate*	−9.1	573.8 ± 46.7	62.7 ± 3.4	−9.01

a The docking affinity was calculated using the Autodock Vina package.

b The obtained value of the mean rupture force *F*_Max_.

c The recorded metric of the pulling work *W*.

d The predicted binding free energy Δ*G*^Pre^_FPL_ was attained using eqn (1). The computed error was the standard error of the average. The unit of energy and force are in kcal mol^−1^ and pN, respectively.

### CPU time consumption

Each SARS-CoV-2 Mpro + ligand complex was simulated over 8 independent FPL simulations, which started from same initial conformation but different random velocity. One FPL trajectory includes 0.1 ns of NVT, 2.0 ns of NPT and 0.5 ns of SMD simulations. 20.8 ns of MD simulations was thus performed to appraise the ligand-binding affinity of the SARS-CoV-2 Mpro with 8 various FPL trajectories. It should be noted that one personal computer with AMD Ryzen 9 3950X CPU and RTX 2060 Super acceleration can perform *ca.* 80 ns of MD simulation per day for the SARS-CoV-2 Mpro + inhibitor system. Therefore, the binding affinity of a ligand to the SARS-CoV-2 Mpro is able to compute 8 times during *ca.* 6.24 hours. The low CPU requirement permits us to rapidly calculate the binding affinity of many ligands to the SARS-CoV-2 Mpro without any professional computing system.

## Conclusions

We have demonstrated that a combination of molecular docking using Autodock Vina and FPL simulations is able to efficiently estimate the binding affinity of a ligand to the SARS-CoV-2 Mpro. In particular, by testing on eleven available inhibitors for preventing the activity of the SARS-CoV-2 Mpro, the computed binding energies were in good agreement with the respective experiments.^11–14^ The correlation coefficient and RMSE are measured as *R*_Dock_ = 0.72 ± 0.14 and RMSE = 2.42 ± 0.22 kcal mol^−1^, respectively. Moreover, the FPL simulations also produced results that are in good agreement with these experiments.^11–14^ The correlation coefficient and RMSE with linear regression are *R*_W_ = −0.76 ± 0.10 and RMSE_W_ = 1.03 ± 0.14 kcal mol^−1^, respectively.

The combination of two approaches is thus employed to predict probable inhibitors for the SARS-CoV-2 Mpro. A short list consisting of sixty-one compounds was found after 36 089 compounds were docked to the binding pocket of the SARS-CoV-2 Mpro. The obtained results were then refined *via* the FPL calculations. Twenty compounds were finally suggested to be able to prevent the activity of the SARS-CoV-2 Mpro because they have low Δ*G*^Pre^_FPL_, which is smaller than −9.00 kcal mol^−1^. Further investigation using *in vitro* and/or *in vivo* studies should be carried out to validate the obtained results.

In addition, as discussed above, the requirement of CPU time is quite low. The combination of the Autodock Vina and FPL simulations are efficient ways for the rapid screening a large number of trial ligands for the SARS-CoV-2 Mpro. In particular, the computations can be carried out at home using an affordable PC with AMD CPU and Nvidia RTX GPU card acceleration.

## Conflicts of interest

There are no conflicts to declare.

## Supplementary Material

RA-010-D0RA06212J-s001
