# Correction: Rapid prediction of possible inhibitors for SARS-CoV-2 main protease using docking and FPL simulations

**DOI:** 10.1039/d2ra90114e

**Published:** 2022-12-15

**Authors:** Minh Quan Pham, Khanh B. Vu, T. Ngoc Han Pham, Le Thi Thuy Huong, Linh Hoang Tran, Nguyen Thanh Tung, Van V. Vu, Trung Hai Nguyen, Son Tung Ngo

**Affiliations:** Graduate University of Science and Technology, Vietnam Academy of Science and Technology Hanoi Vietnam; Institute of Natural Products Chemistry, Vietnam Academy of Science and Technology Hanoi Vietnam; School of Biotechnology, International University Ho Chi Minh City Vietnam; Vietnam National University Ho Chi Minh City Vietnam; Faculty of Pharmacy, Ton Duc Thang University Ho Chi Minh City Vietnam; Faculty of Civil Engineering, Ho Chi Minh University of Technology (HCMUT) Ho Chi Minh Vietnam; Institute of Materials Science, Vietnam Academy of Science and Technology Hanoi Vietnam; NTT Hi-Tech Institute, Nguyen Tat Thanh University Ho Chi Minh City Vietnam; Laboratory of Theoretical and Computational Biophysics, Ton Duc Thang University Ho Chi Minh City Vietnam ngosontung@tdtu.edu.vn; Faculty of Applied Sciences, Ton Duc Thang University Ho Chi Minh City Vietnam

## Abstract

Correction for ‘Rapid prediction of possible inhibitors for SARS-CoV-2 main protease using docking and FPL simulations’ by Minh Quan Pham *et al.*, *RSC Adv.*, 2020, **10**, 31991–31996, https://doi.org/10.1039/D0RA06212J.

In the original article at the time of publication, ref. 44 had only been uploaded to ChemRXiv (DOI: https://doi.org/10.26434/chemrxiv.12771068.v1) and had not been published in a finalised format, so the full reference information was not included. The research described in ref. 44 has been published, and the full details of this reference are included below:

44 S. T. Ngo, H. M. Nguyen, L. T. Thuy Huong, P. M. Quan, V. K. Truong, N. T. Tung and V. V. Vu, Assessing potential inhibitors of SARS-CoV-2 main protease from available drugs using free energy perturbation simulations, *RSC Adv.*, 2020, **10**, 40284–40290.

The Royal Society of Chemistry apologises for these errors and any consequent inconvenience to authors and readers.

## Supplementary Material

