# Supplementary material for: Rapid prediction of possible inhibitors for SARS-CoV-2 main protease using docking and FPL simulations
Source: RSC Adv. 2020 Aug 28;10(53):31991–6. doi: 10.1039/d0ra06212j (PMC9056572; doi:10.1039/d0ra06212j)
Supplement: RA-010-D0RA06212J-s001 [file RA-010-D0RA06212J-s001.pdf]

Electronic Supplementary Material (ESI) for RSC Advances.
This journal is © The Royal Society of Chemistry 2020

Supporting Information

Rapid Prediction of Possible Inhibitors for SARS-CoV-2 Main Protease using Docking and FPL Simulations

Minh Quan Pham,[ab] Khanh B. Vu,[cd] T. Ngoc Han Pham,[e] Le Thi Thuy Huong,[ab] Linh Hoang Tran,[df] Nguyen Thanh Tung,[ag] Van V. Vu,[h] and Trung Hai Nguyen[ij] Son Tung Ngo,[ij]*

[a]Graduate University of Science and Technology, Vietnam Academy of Science and Technology, Hanoi, Vietnam

[b]Institute of Natural Products Chemistry, Vietnam Academy of Science and Technology, Hanoi, Vietnam

[c]School of Biotechnology, International University, Ho Chi Minh City, Vietnam

[d]Vietnam National University, Ho Chi Minh City, Vietnam

[e]Faculty of Pharmacy, Ton Duc Thang University, Ho Chi Minh City, Vietnam

[f]Faculty of Civil Energeering, Ho Chi Minh University of Technology (HCMUT), Ho Chi Minh, Vietnam

[g]Institute of Materials Science, Vietnam Academy of Science and Technology, Hanoi, Vietnam

[h]NTT Hi-Tech Institute, Nguyen Tat Thanh University, Ho Chi Minh City, Vietnam

[i]Laboratory of Theoretical and Computational Biophysics, Ton Duc Thang University, Ho Chi Minh City, Vietnam

[j]Faculty of Applied Sciences, Ton Duc Thang University, Ho Chi Minh City, Vietnam

*Email: ngosontung@tdtu.edu.vn

Table 1. Comparison of the docking results of available inhibitors on the SARS-CoV-2 monomer and dimer.

N^0	Name	$\Delta G_{\mathrm{Dock}}^{\mathrm{Monomer}}$	$\Delta G_{\mathrm{Dock}}^{\mathrm{Dimer}}$
1	**11r**	-6.5	-7.0
2	**13a**	-6.5	-6.3
3	**13b**	-6.3	-7.3
4	**11a**	-6.8	-7.3
5	**11b**	-7.0	-7.7
6	*Carmofur*	-5.7	-5.9
7	*Disulfiram*	-3.8	-4.2
8	*Ebselen*	-5.6	-6.2
9	*PX-12*	-3.8	-4.1
10	*Shikonin*	-6.1	-6.9
11	*Tideglusib*	-6.6	-6.6

The unit is in kcal mol^{-1}.

Table S2. The Protein-Ligand Interaction Maps from Docking Conformation.

**11a**<br>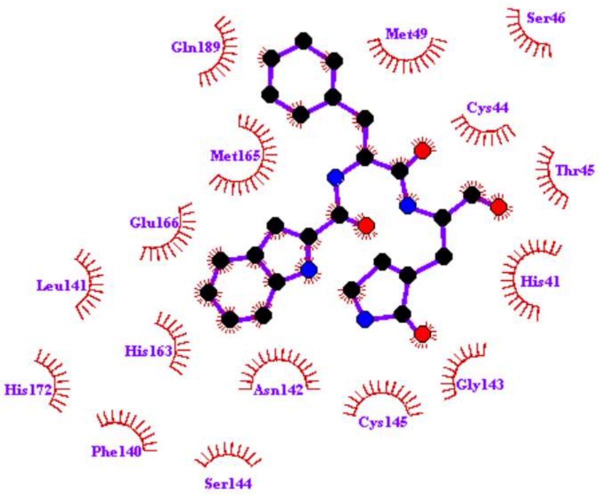 	**11b**<br>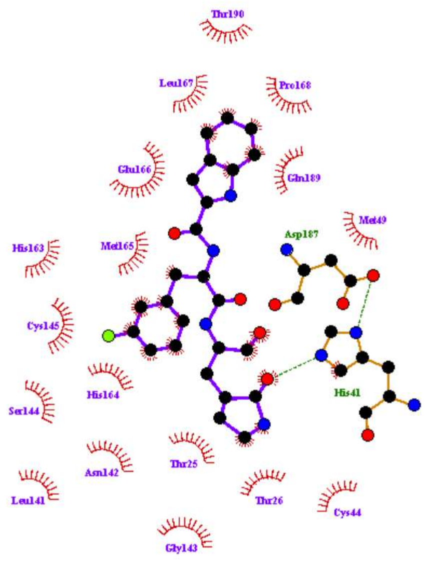
**11r**<br>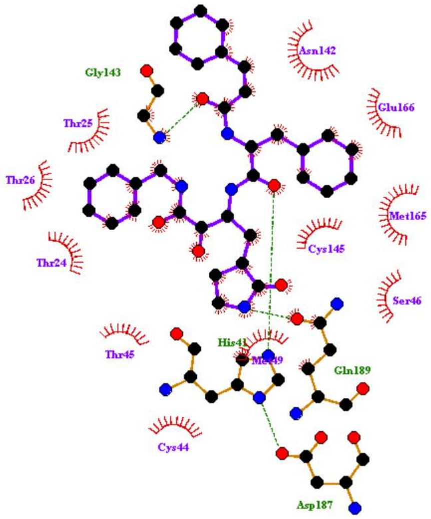 	**13a**<br>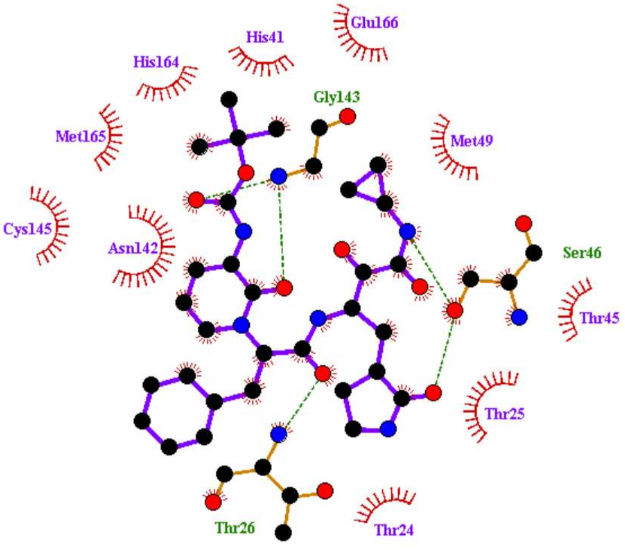

13b

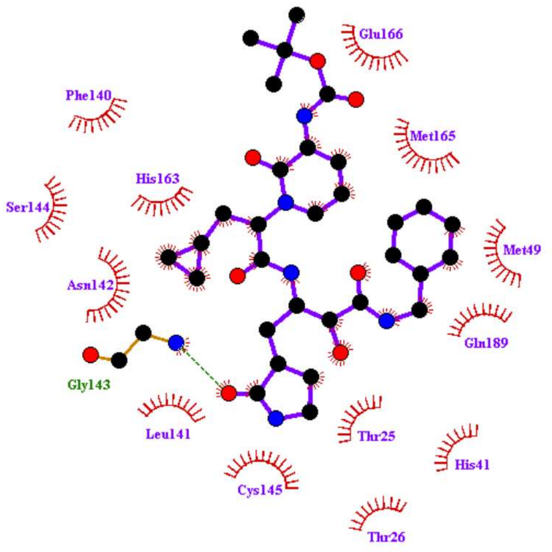

Carmofur

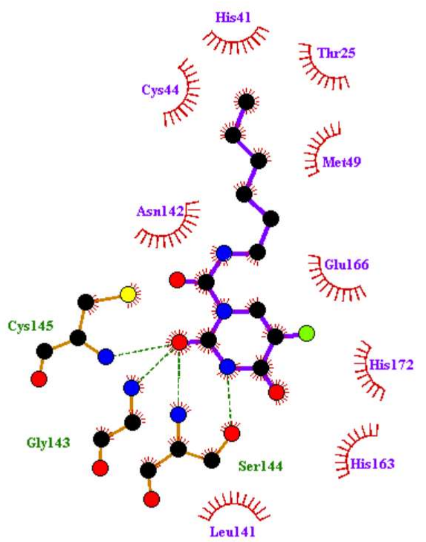

Disulfiram

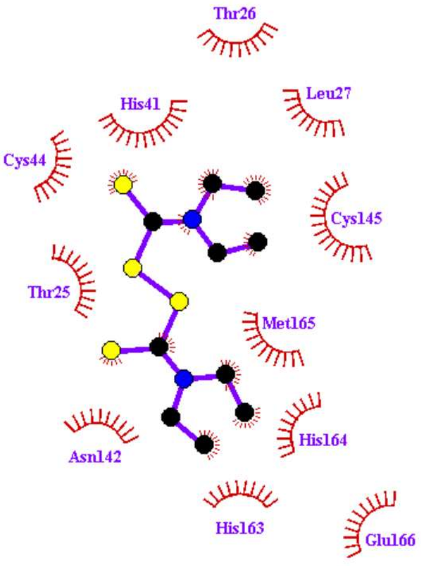

Ebselen

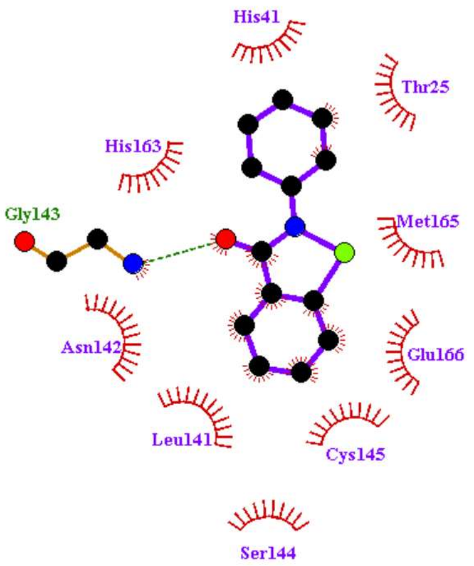

PX-12

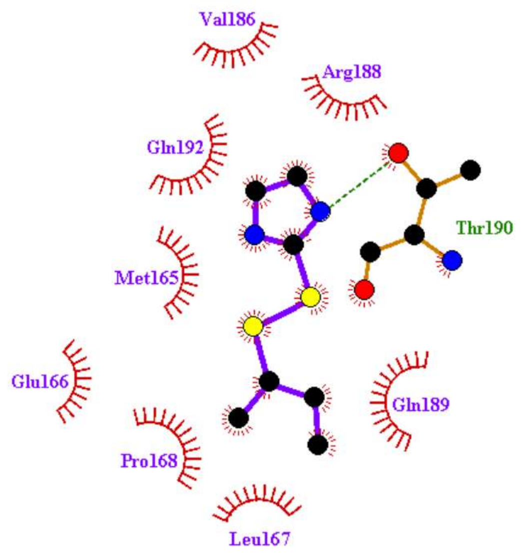

Shikonin

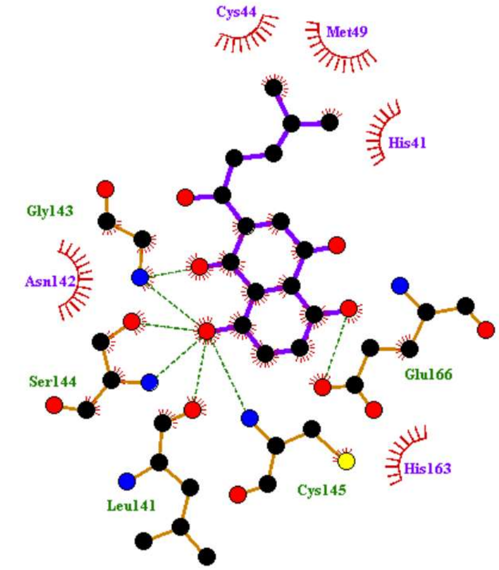

Tideglusib

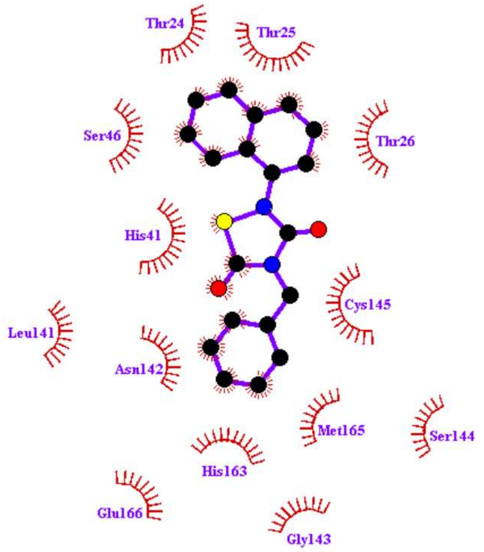

ZINC000256110404

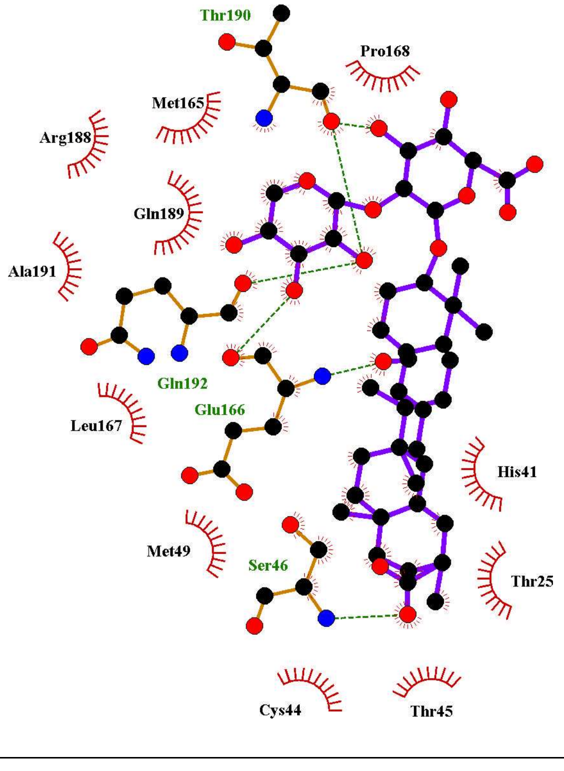

ZINC000085537131

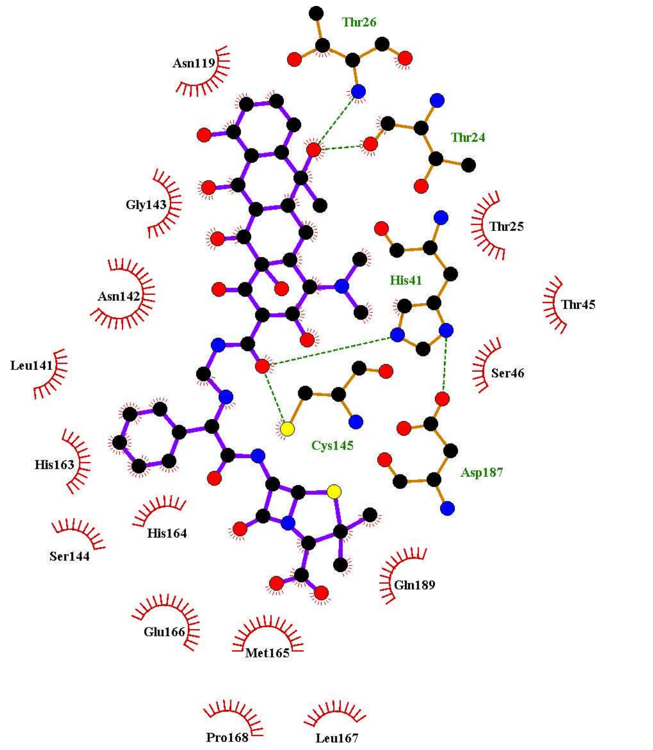

ZINC000100783644

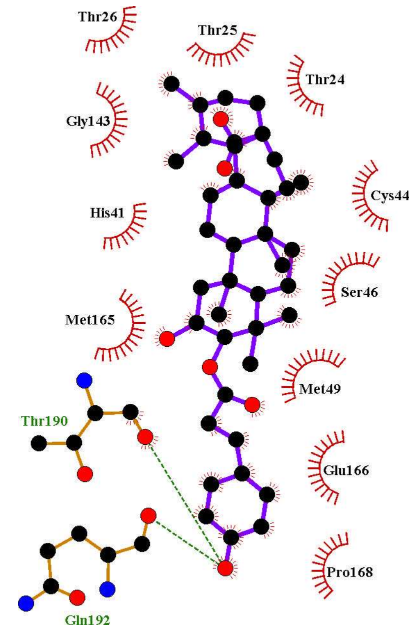

ZINC000253527863

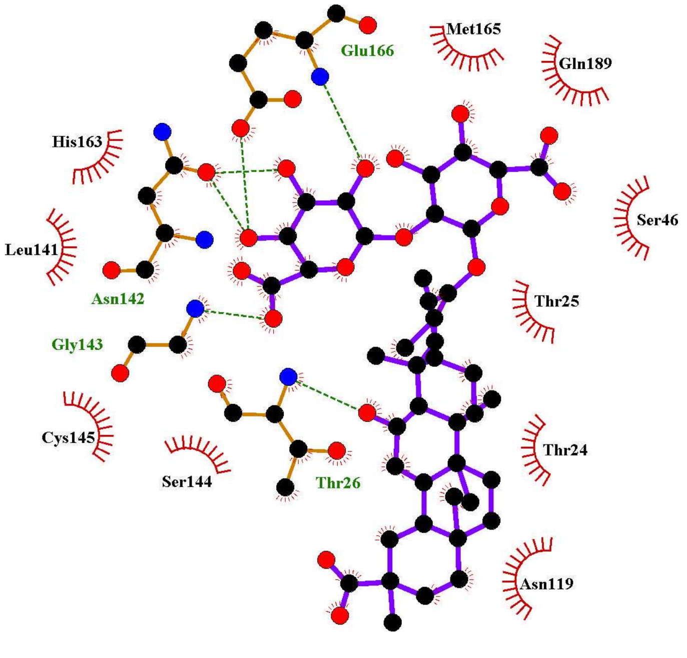

ZINC000256105139

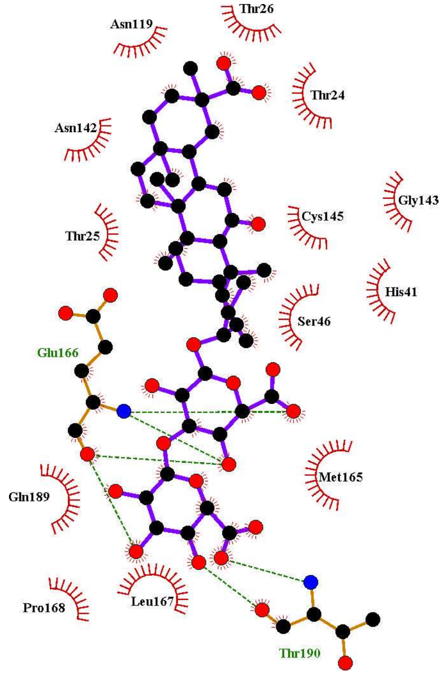

ZINC000100783815

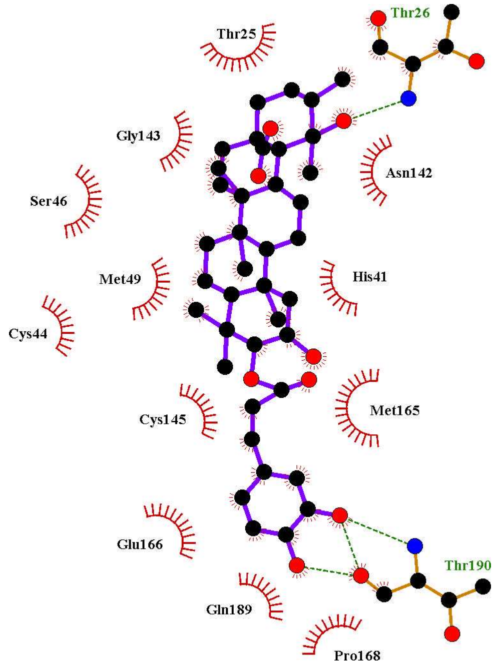

ZINC000004214527

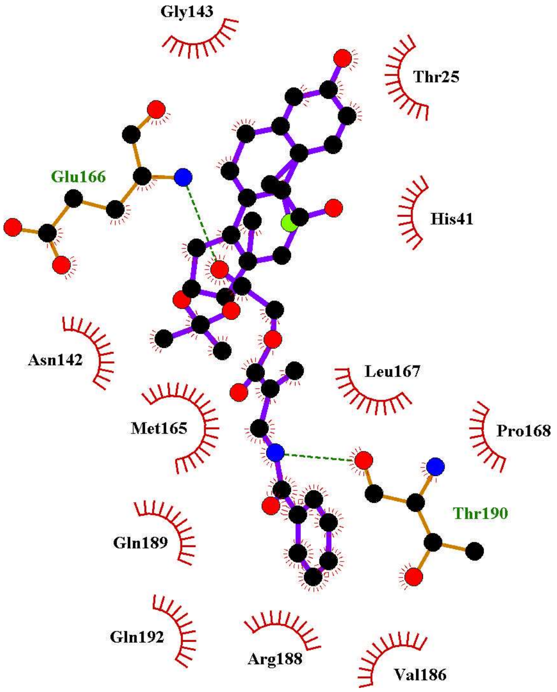

ZINC000028642721

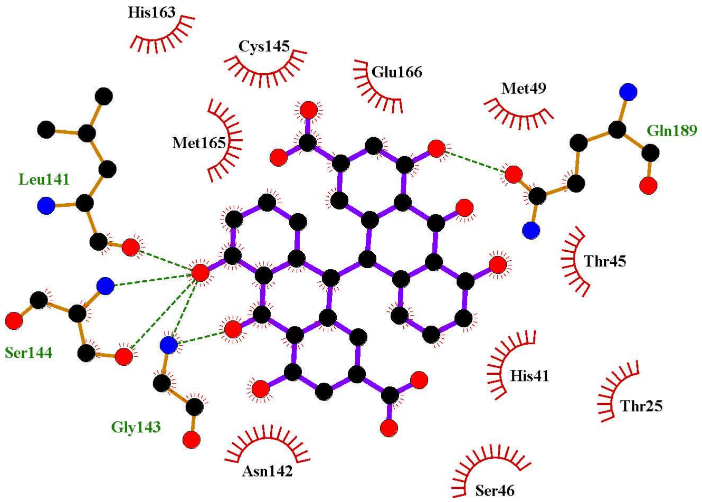

ZINC000100783890

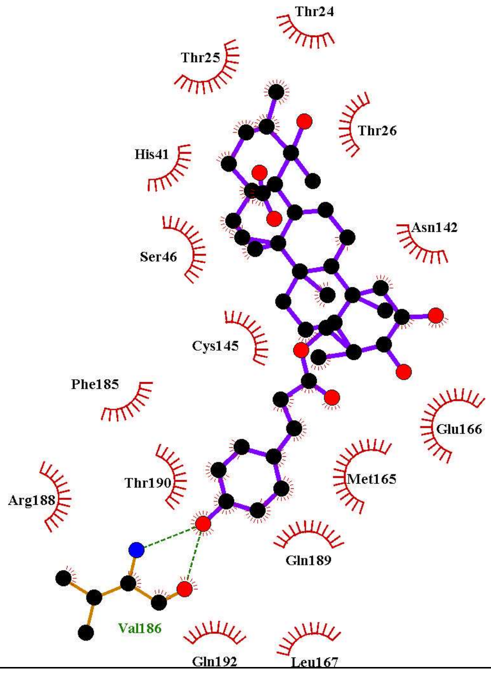

ZINC000098052857

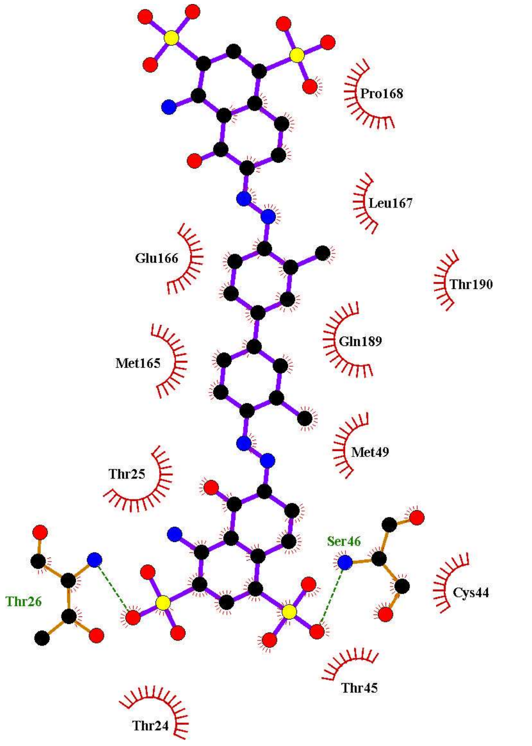

ZINC000100783691

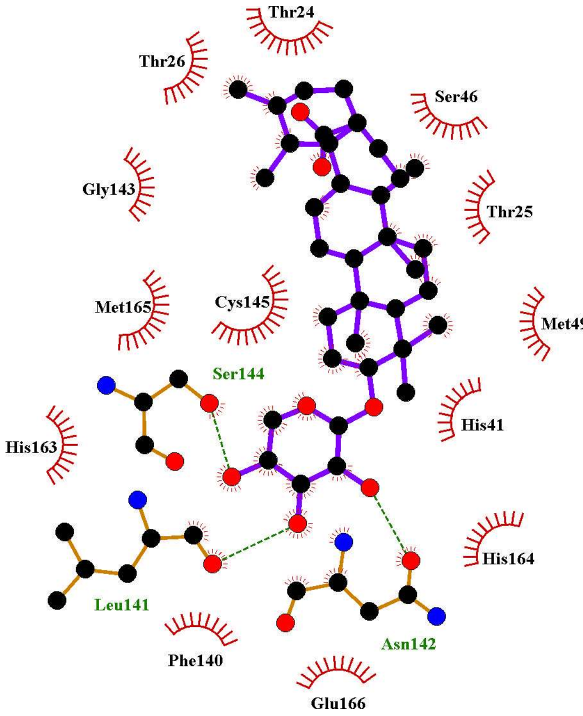

ZINC000095619992

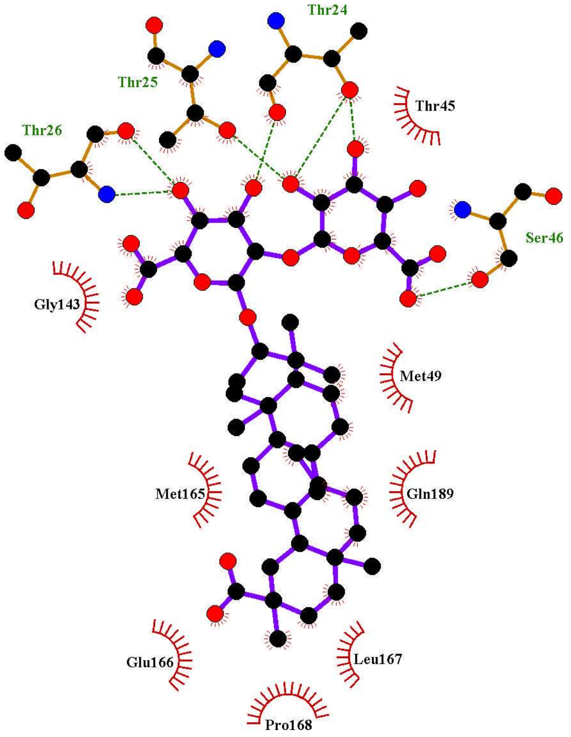

ZINC000118937488

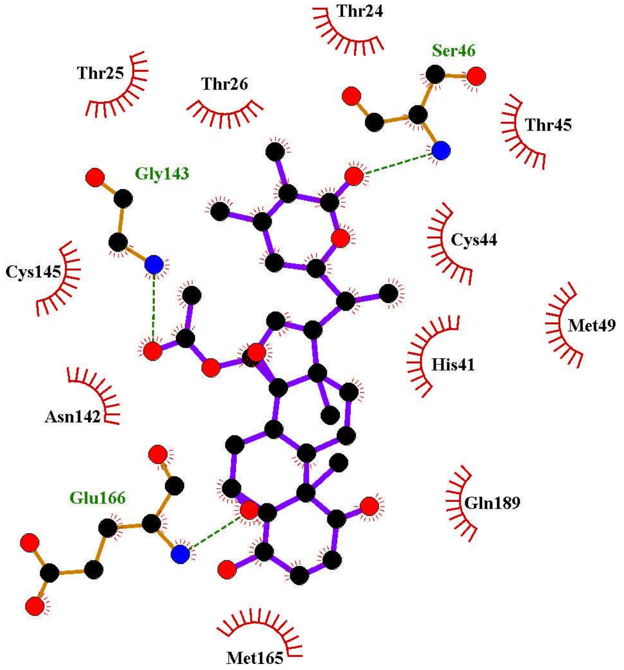

ZINC000100783660

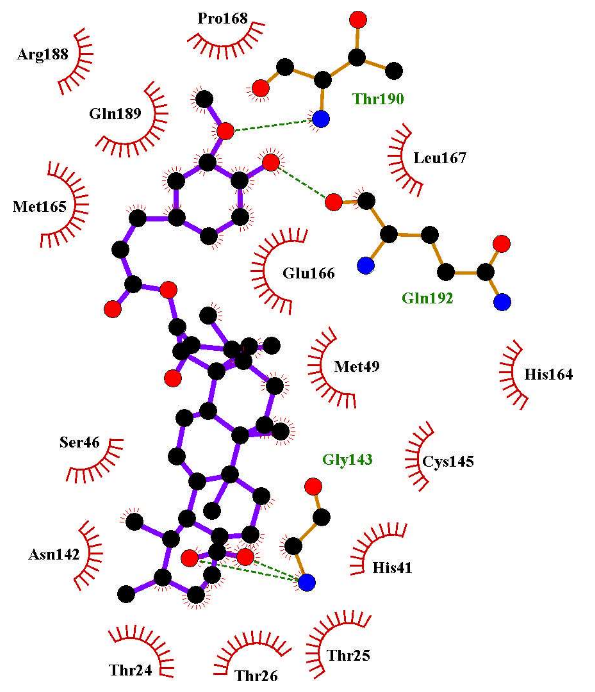

ZINC000100777487

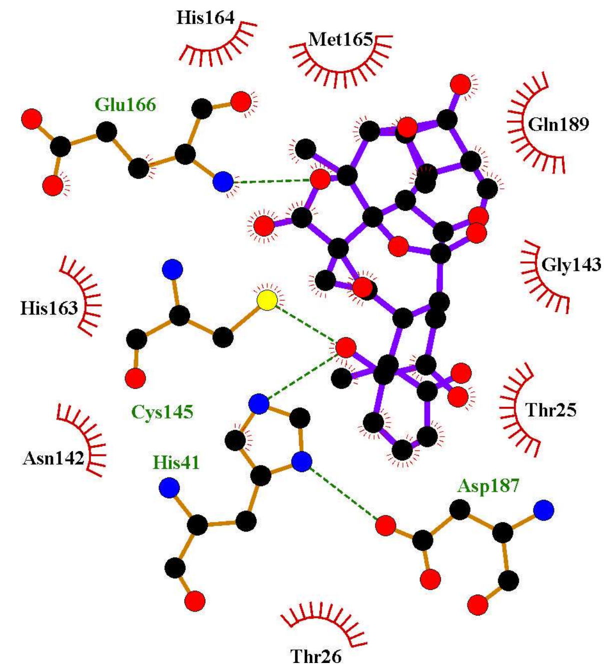

ZINC000004879678

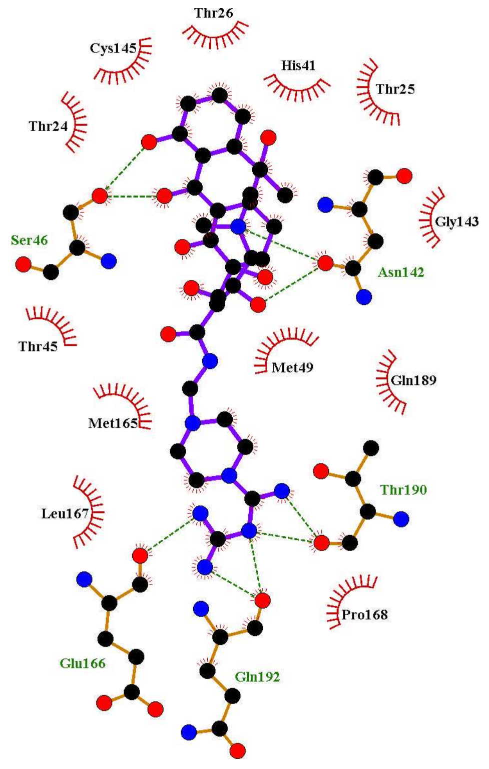

ZINC000150354128

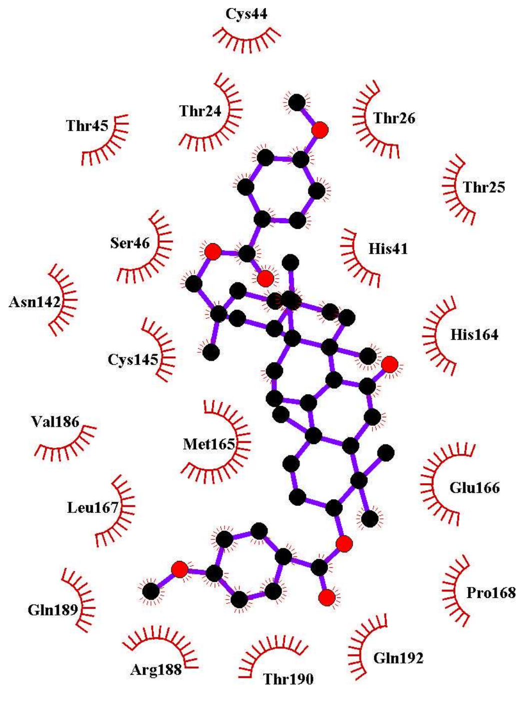

ZINC000004215464

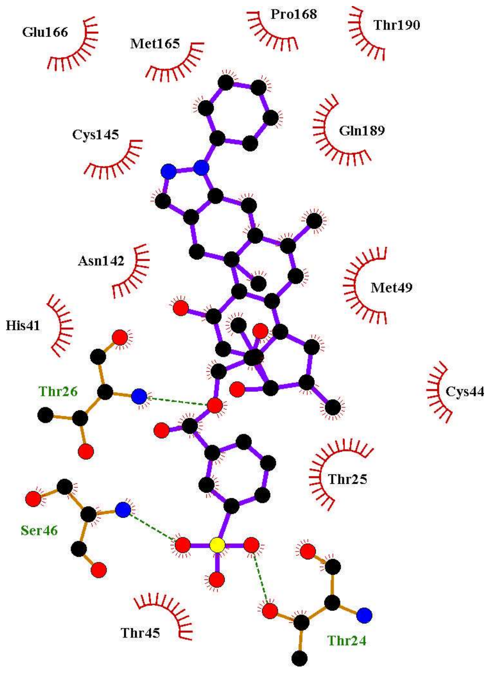

ZINC000100774273

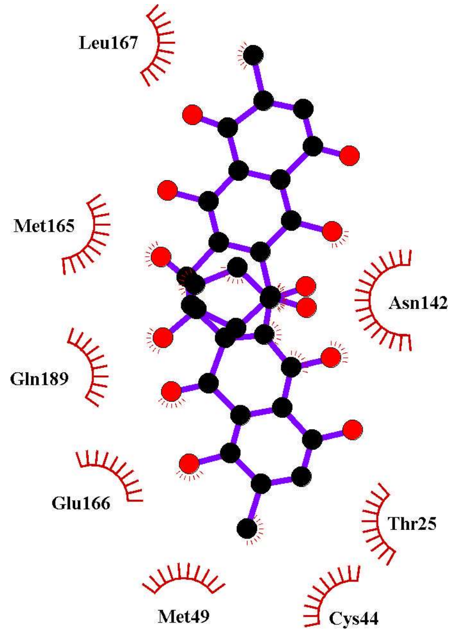

ZINC000073224787

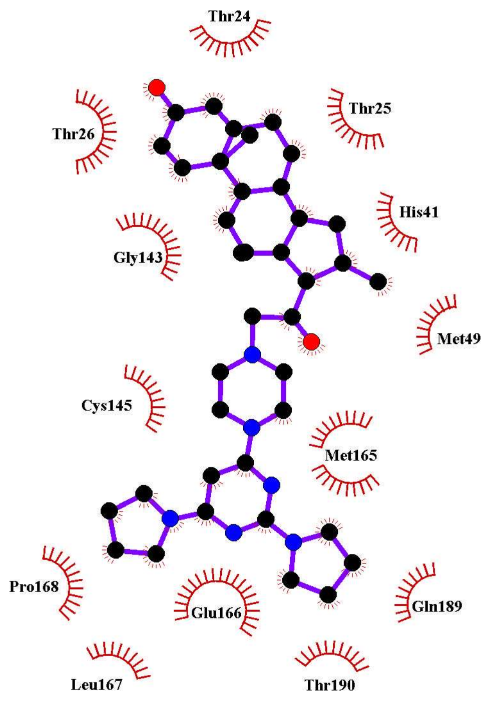

ZINC000253599724

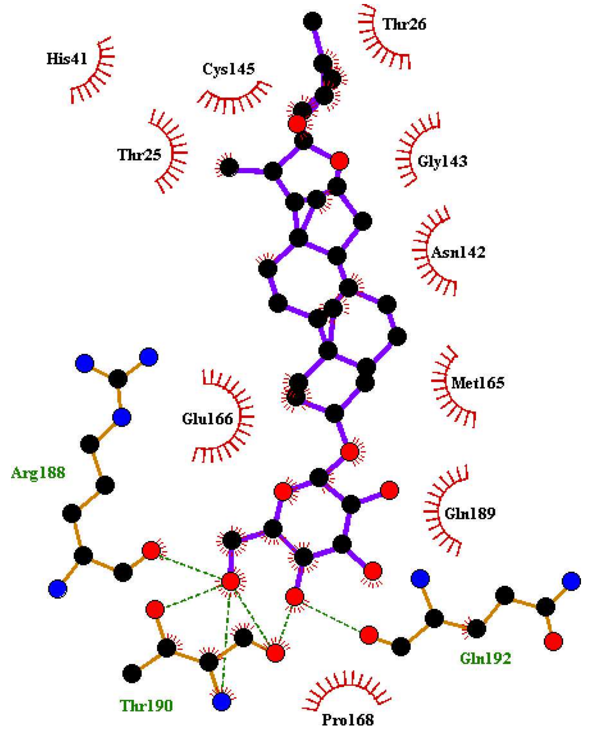

ZINC000100774275

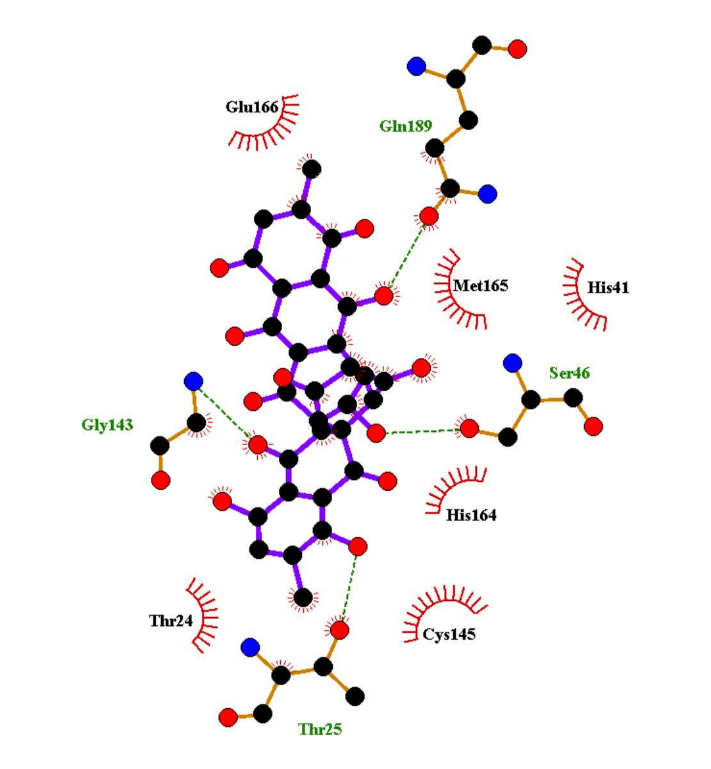

ZINC000004215844

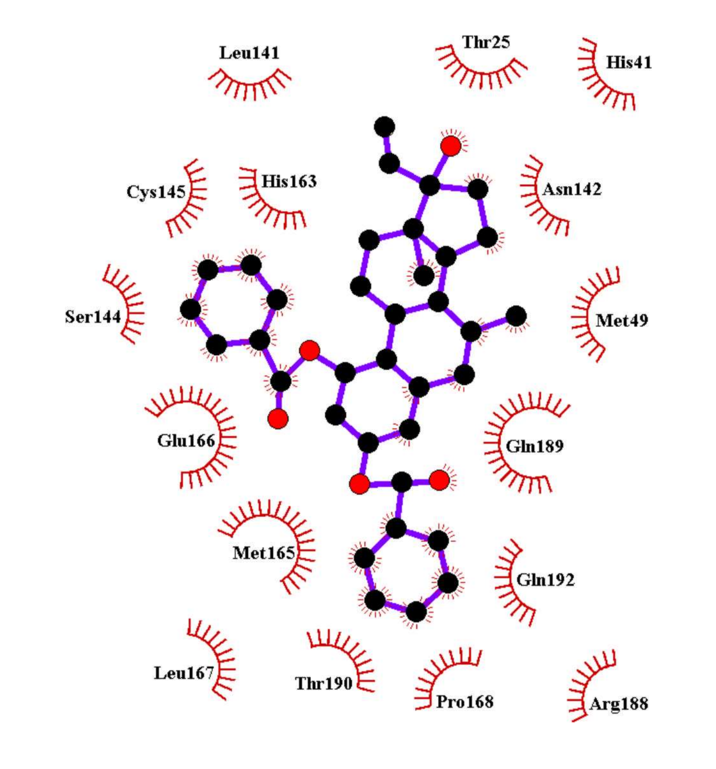

ZINC000085537129

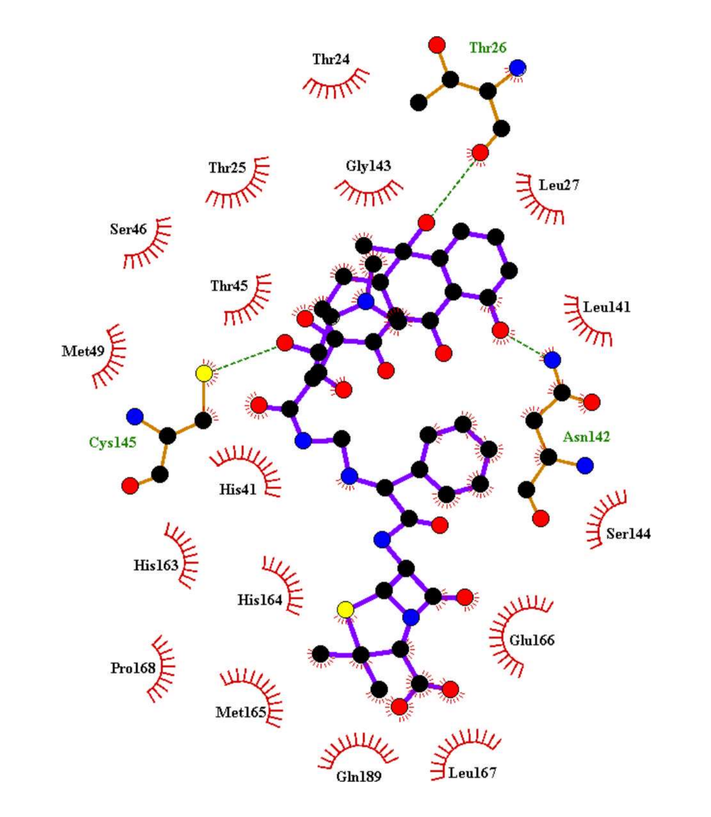

ZINC000100774279

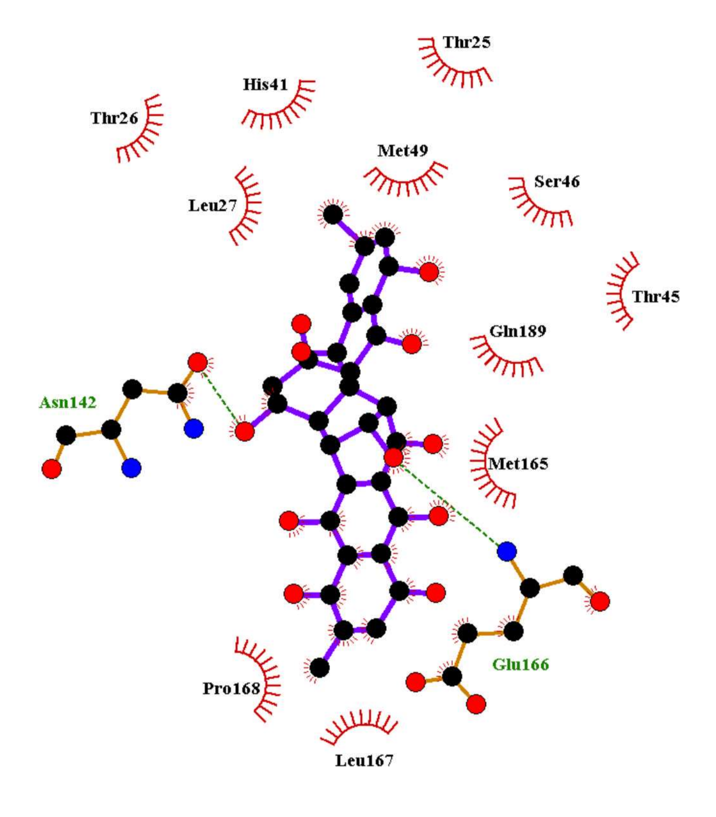

ZINC000100777465

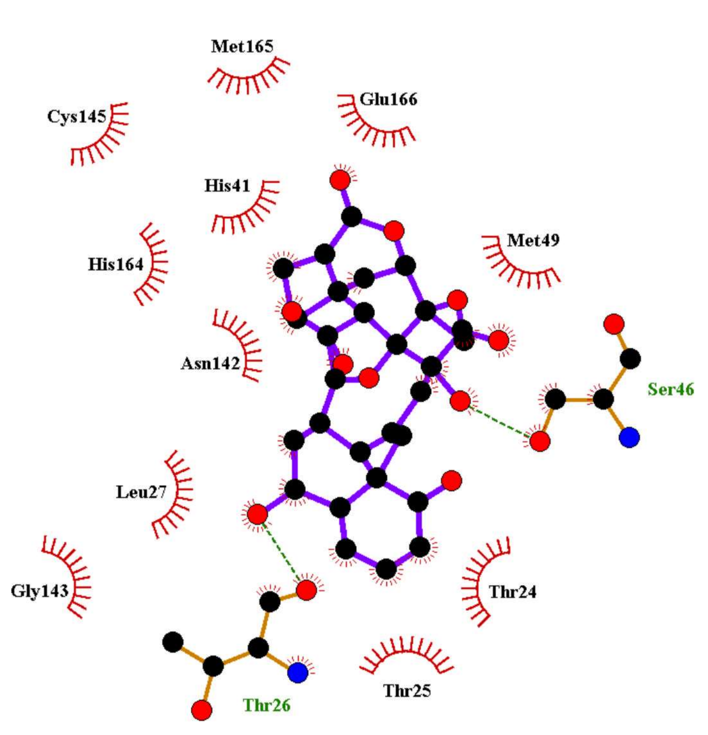

ZINC000113198271

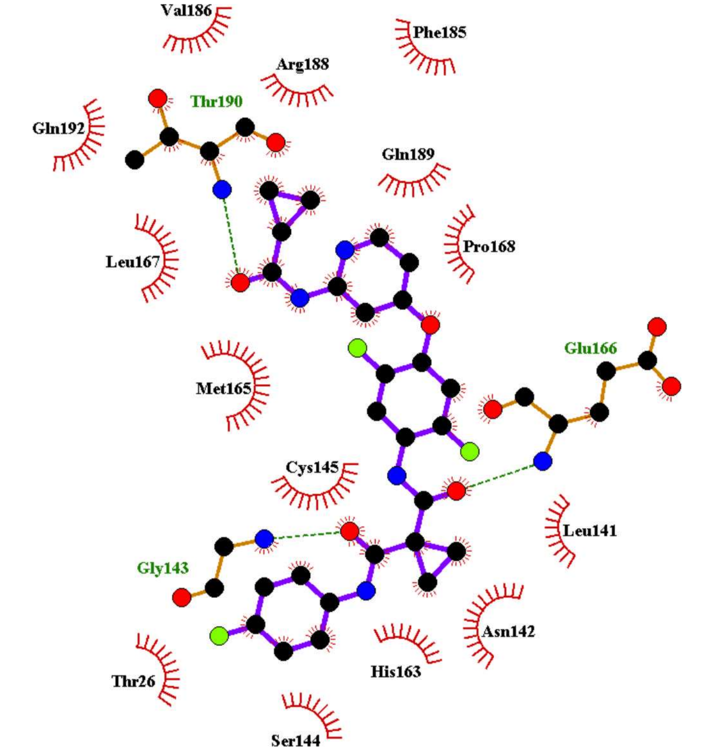

ZINC000085488845

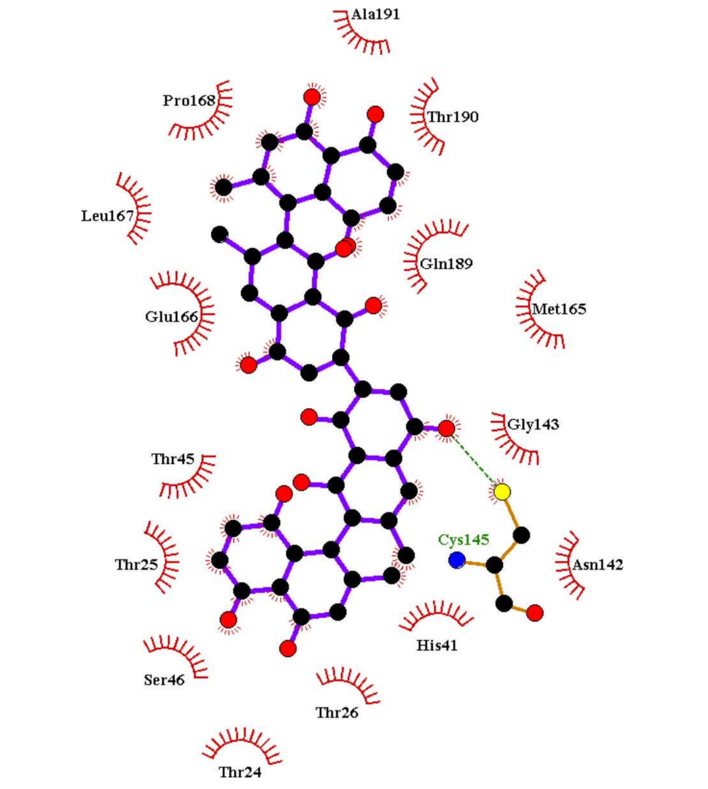

ZINC000095564635

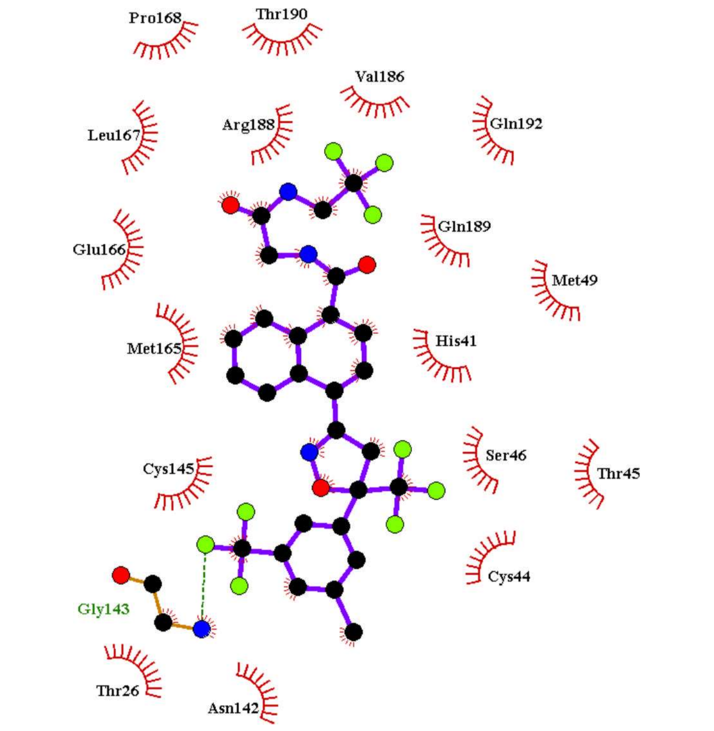

ZINC000100772783

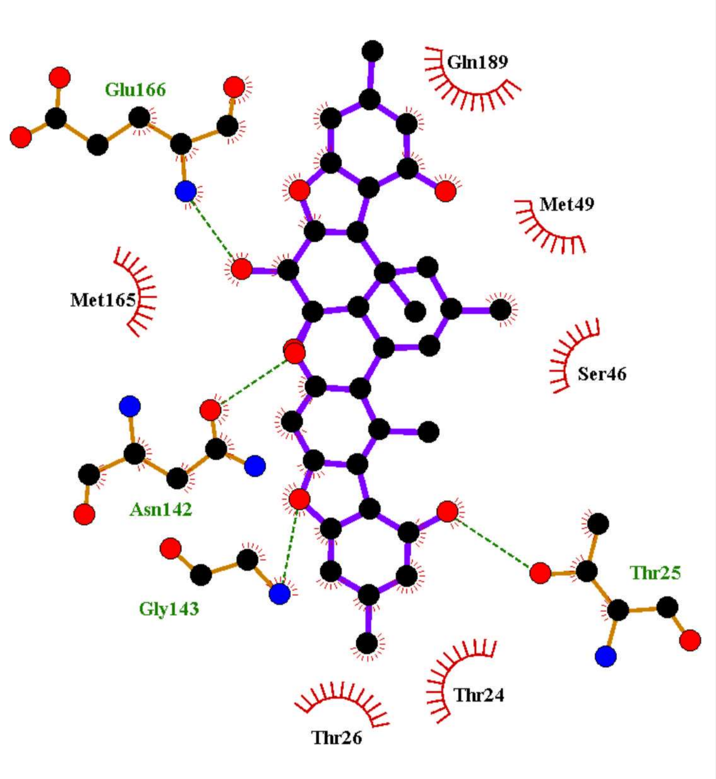

ZINC000100826921

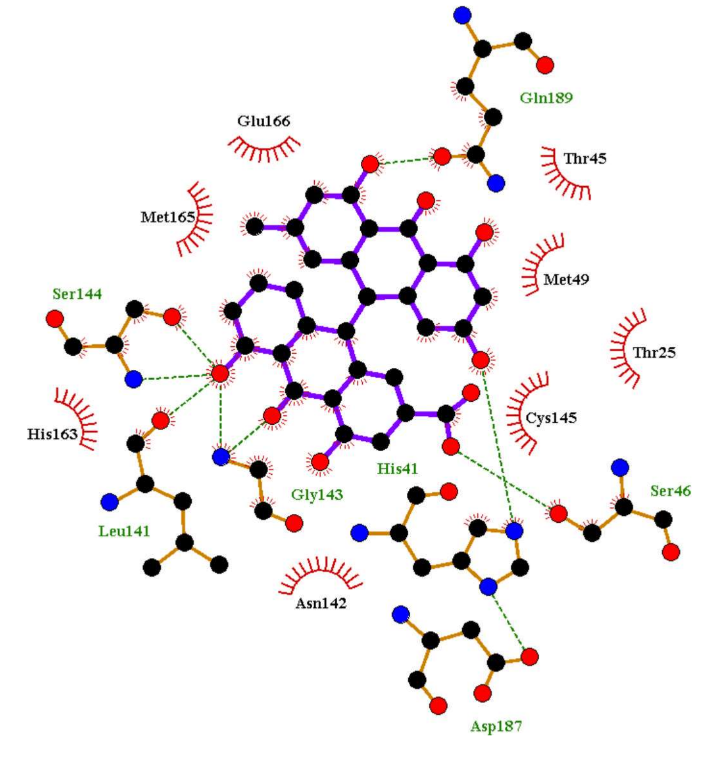

ZINC000051951668

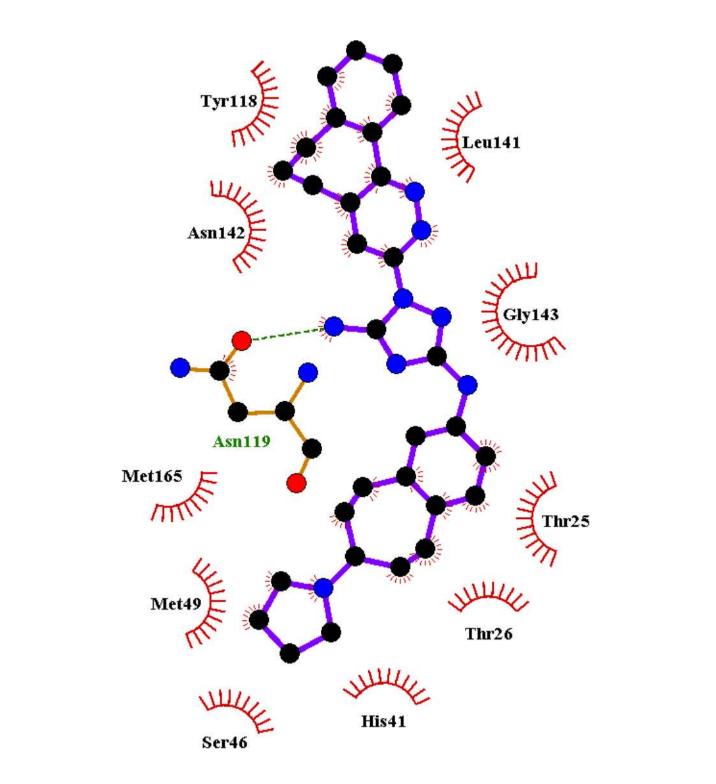

ZINC000100822646

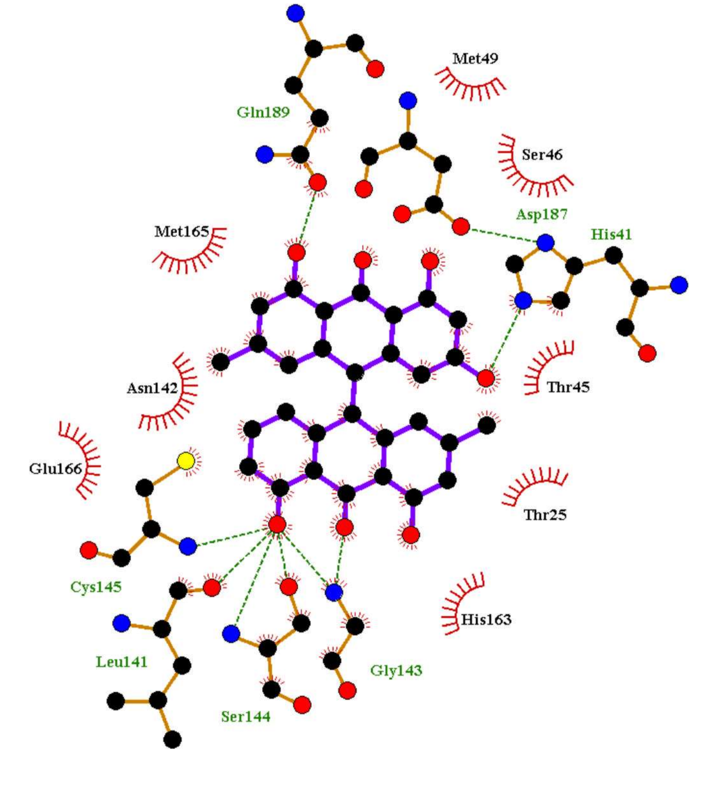

ZINC000004214742

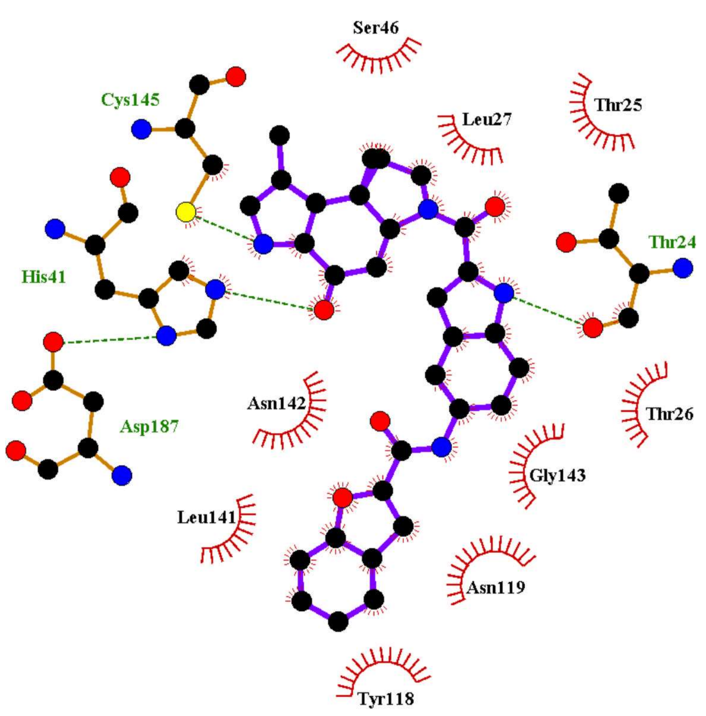

ZINC000239329011

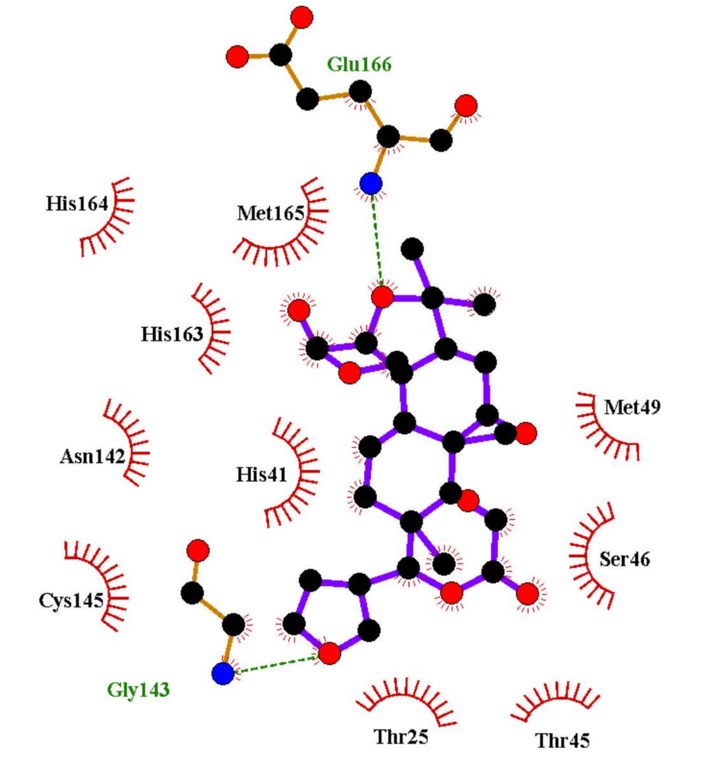

ZINC000118937393

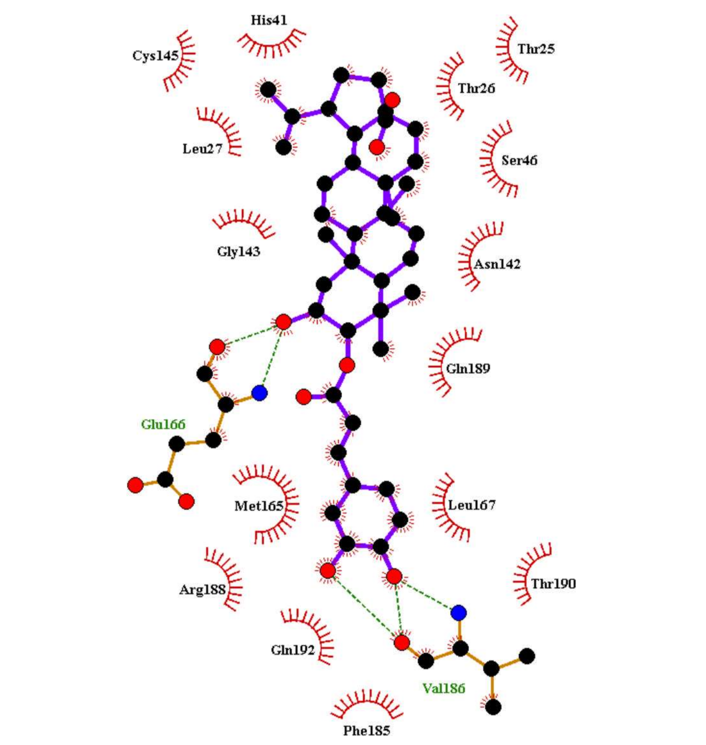

ZINC000103941727

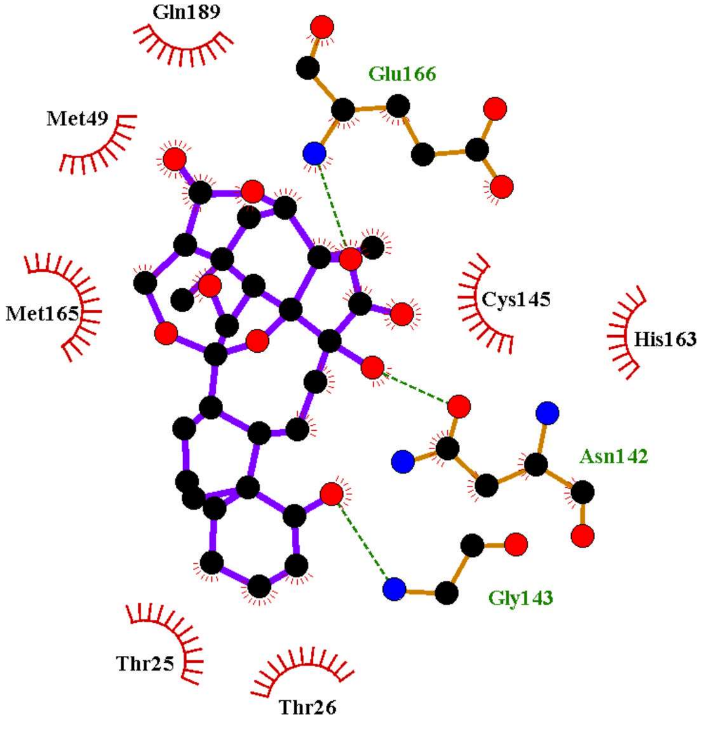

ZINC000043061730

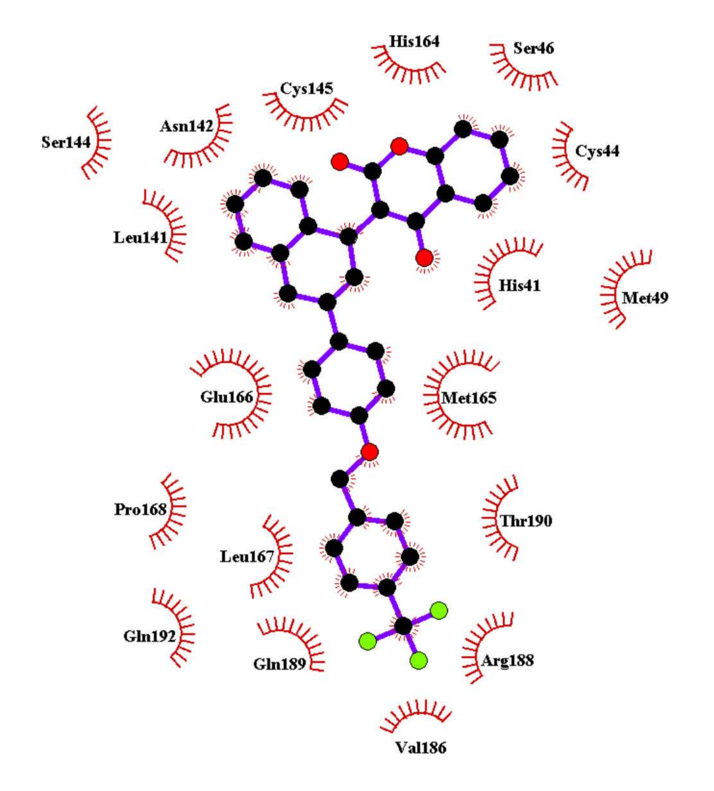

ZINC000256421903

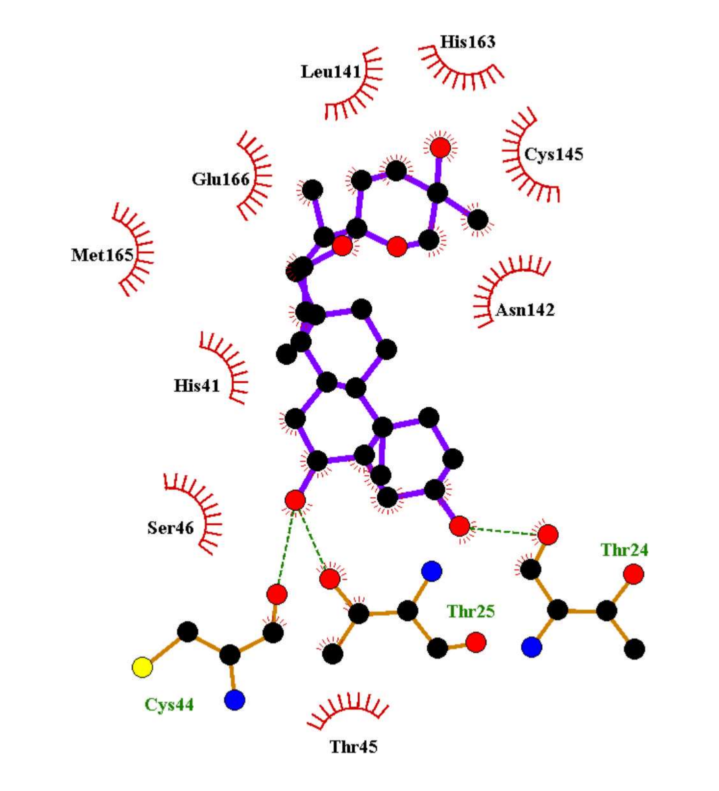

ZINC000150353464

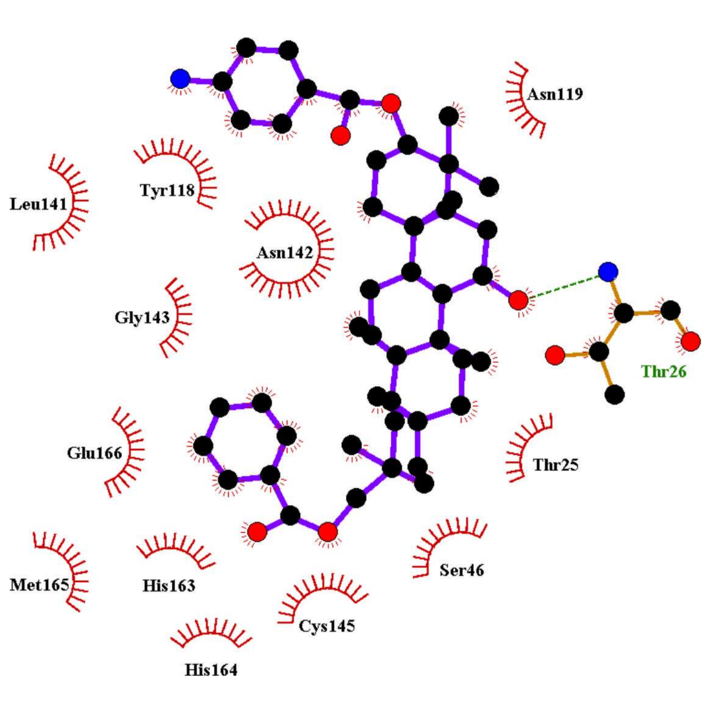

ZINC000253527861

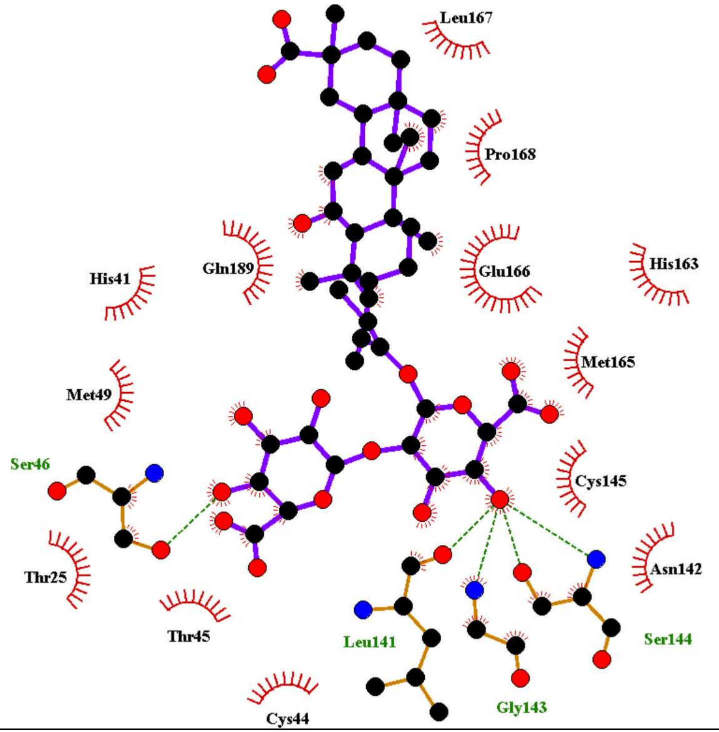

ZINC000100772795

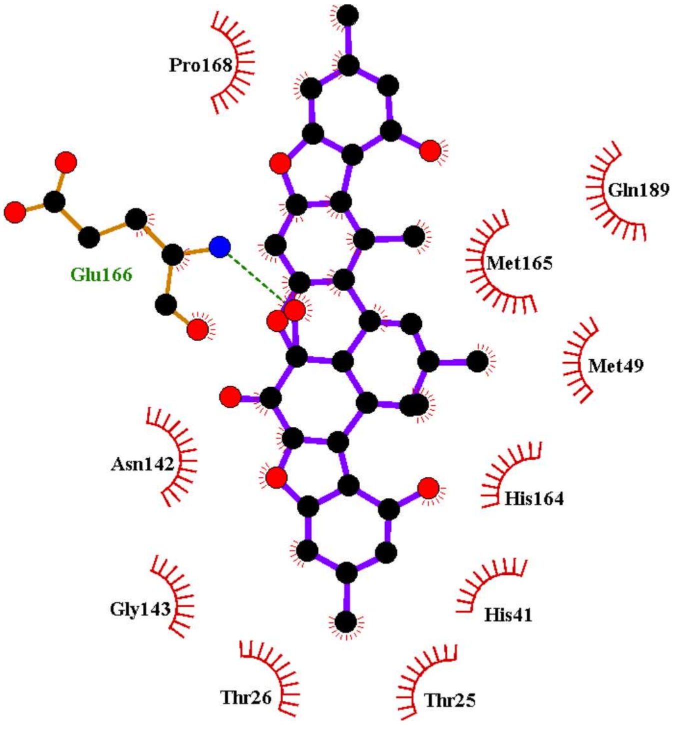

ZINC000095619883

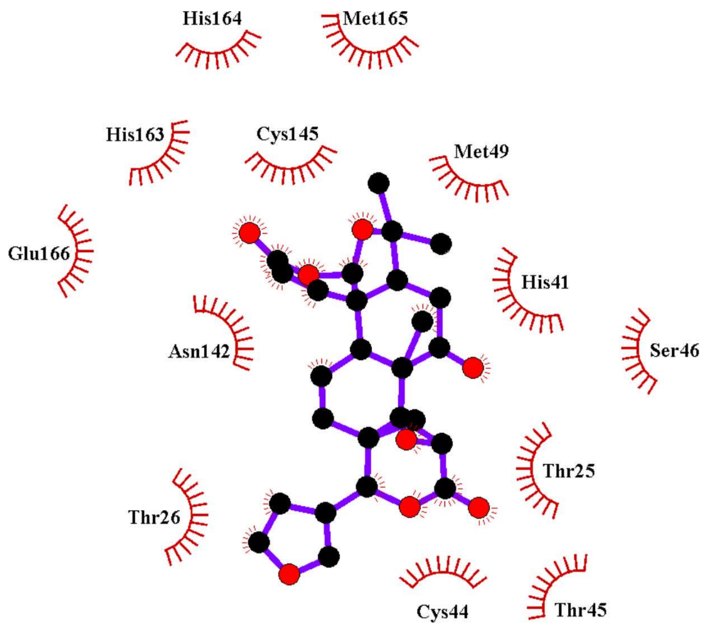

ZINC000100822774

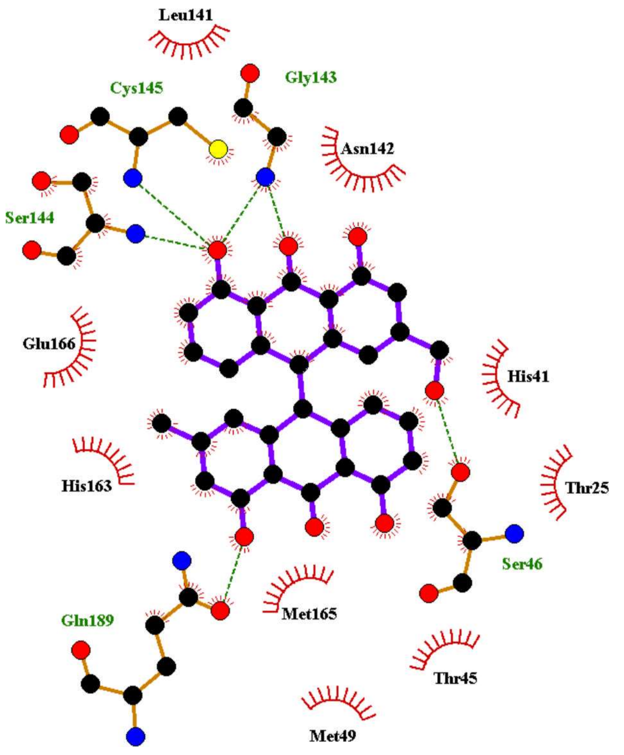

ZINC000100783375

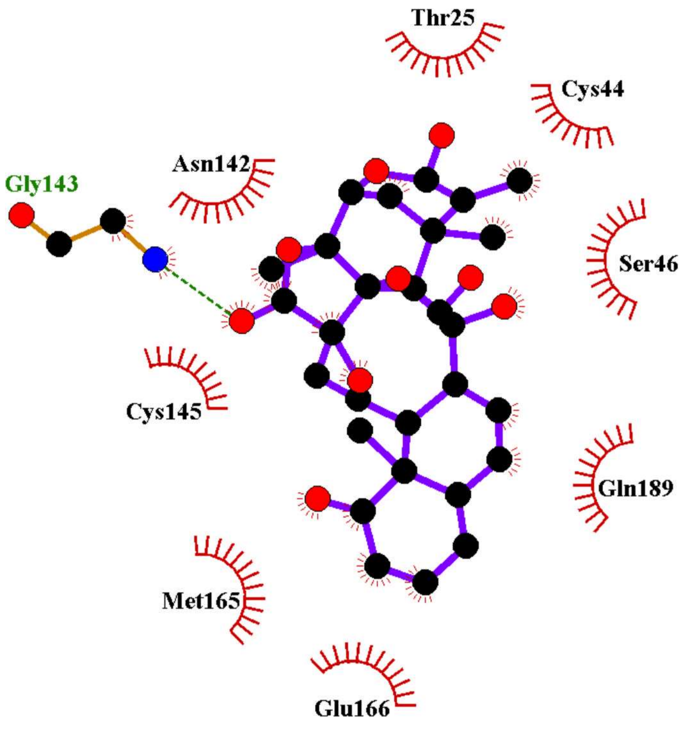

ZINC000100783373

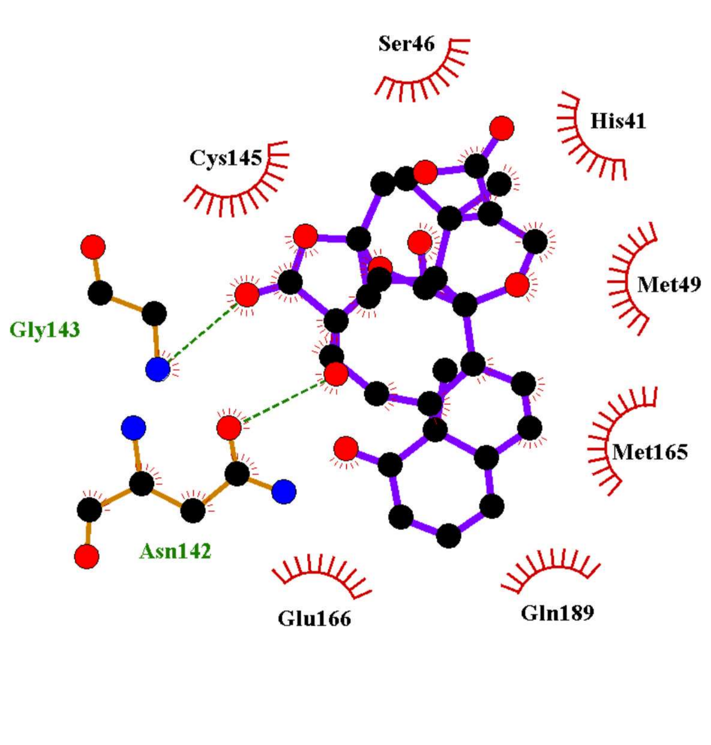

ZINC000261496785

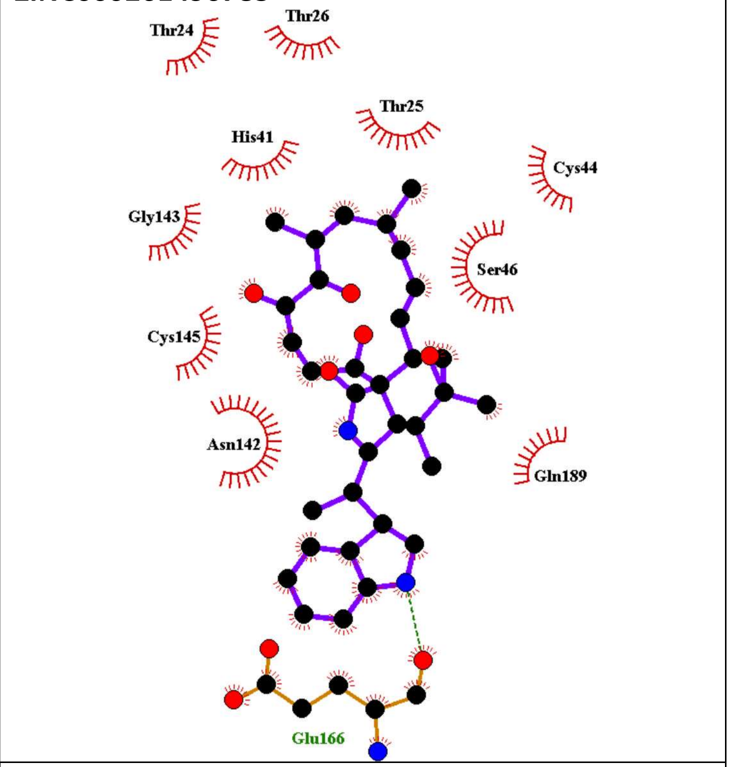

ZINC000100826926

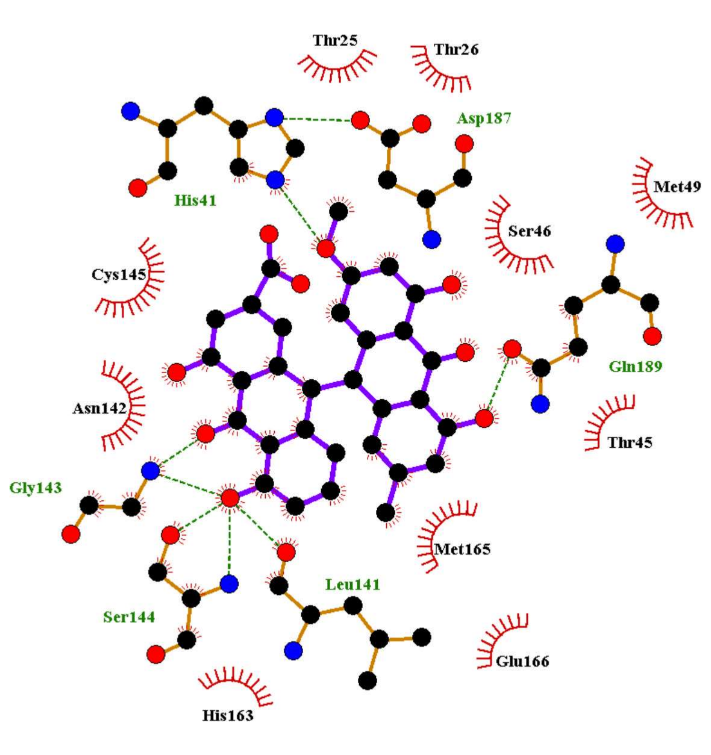

ZINC000100777456

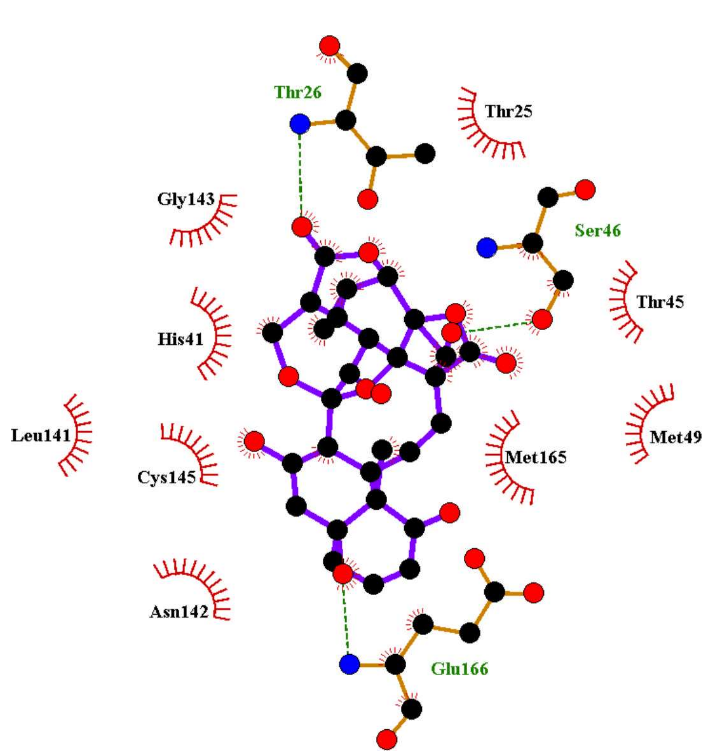

ZINC000100779057

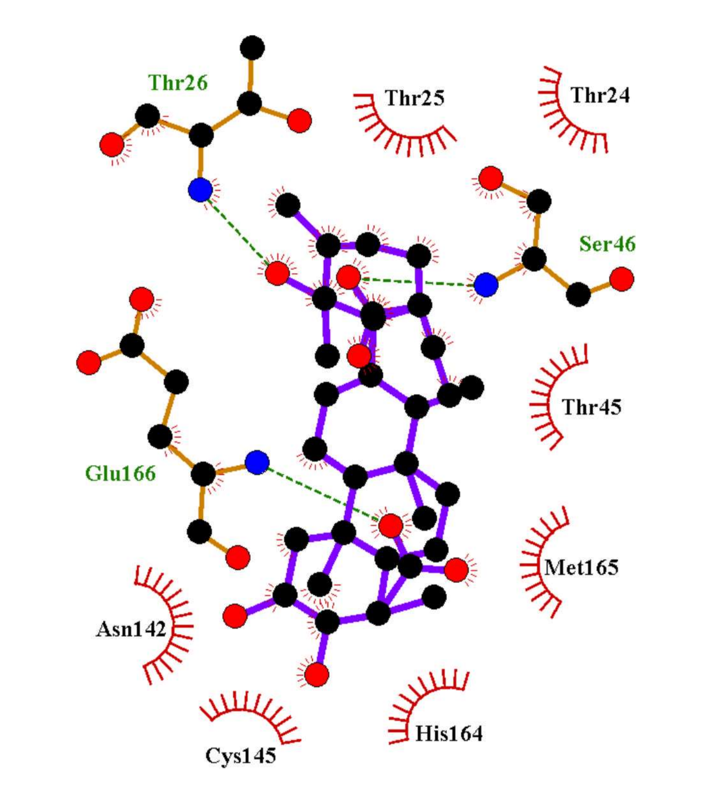

ZINC000118937472

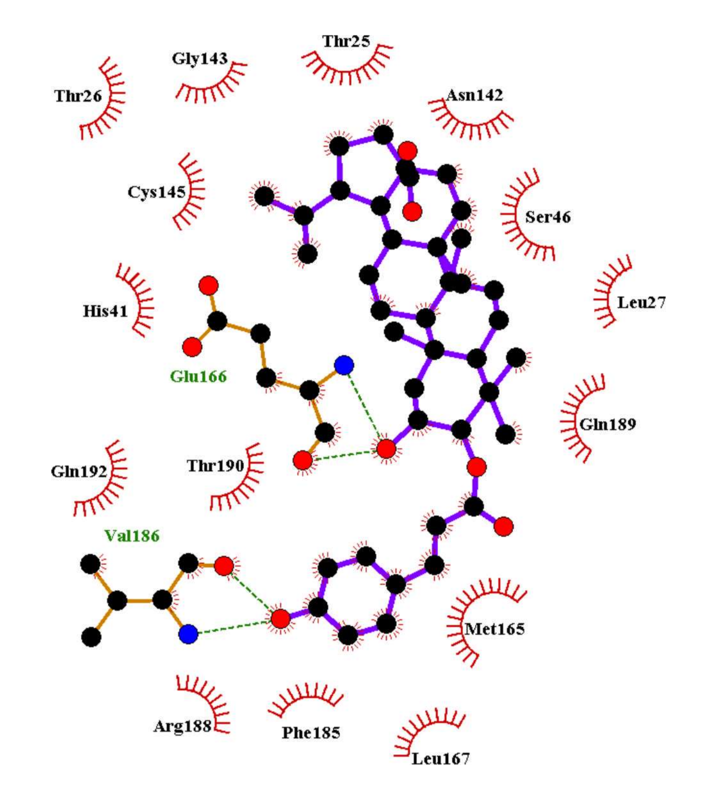

ZINC000100783384

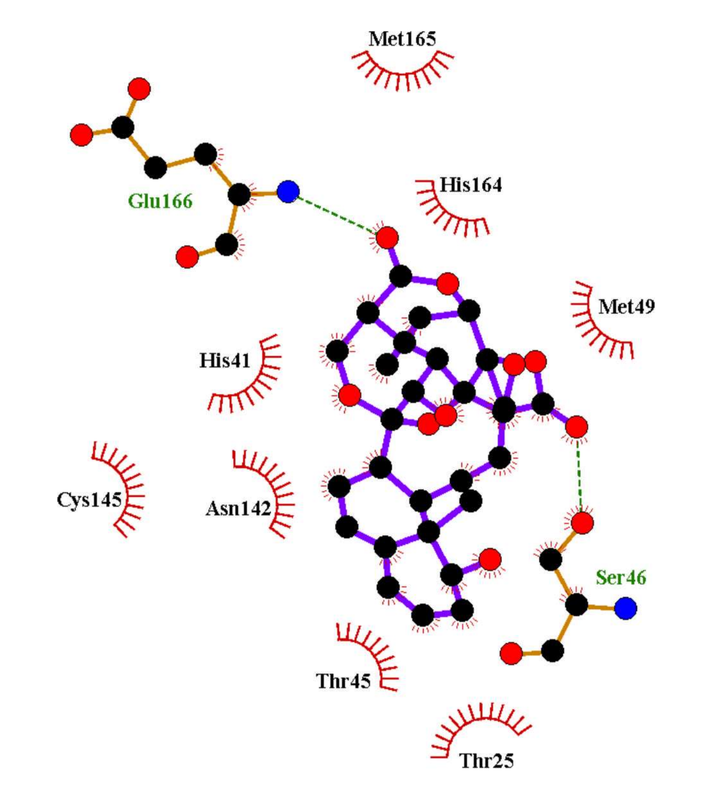

ZINC000100777392

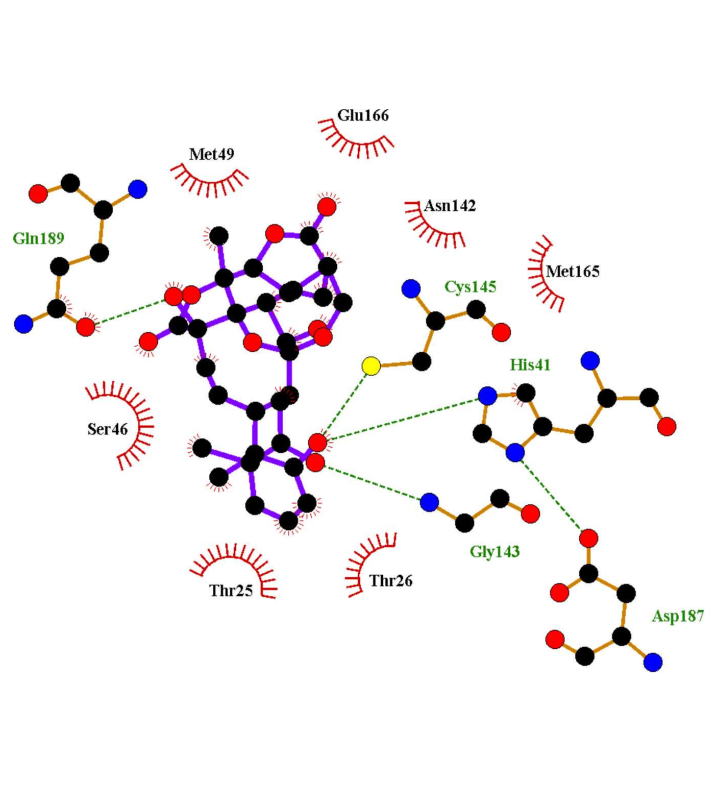

ZINC000100782938

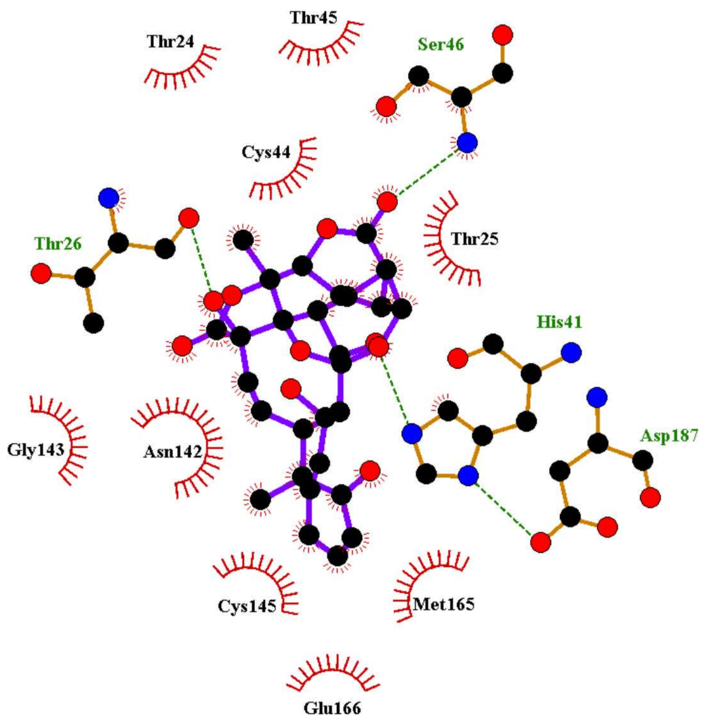

ZINC000150353094

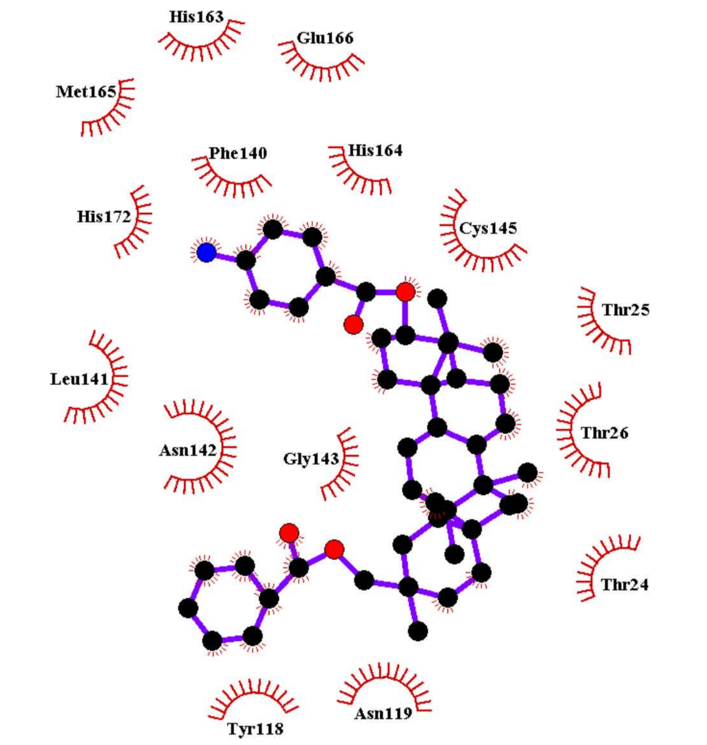

ZINC000100783281

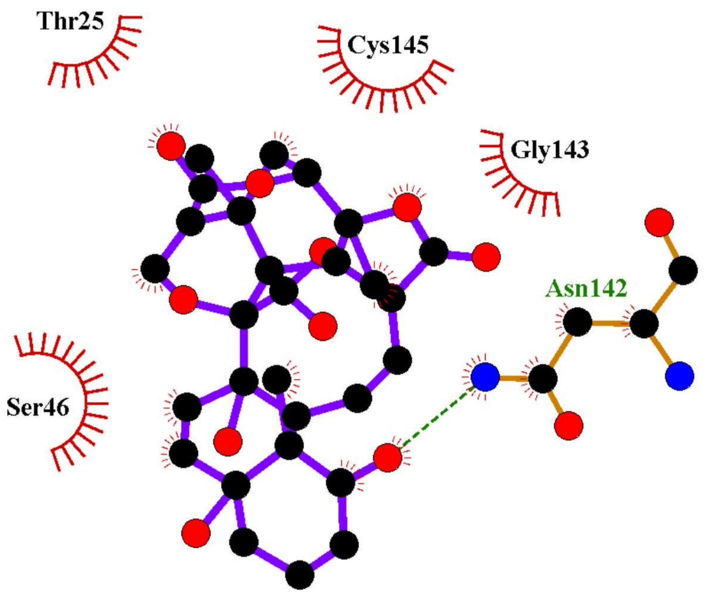

ZINC000070450821

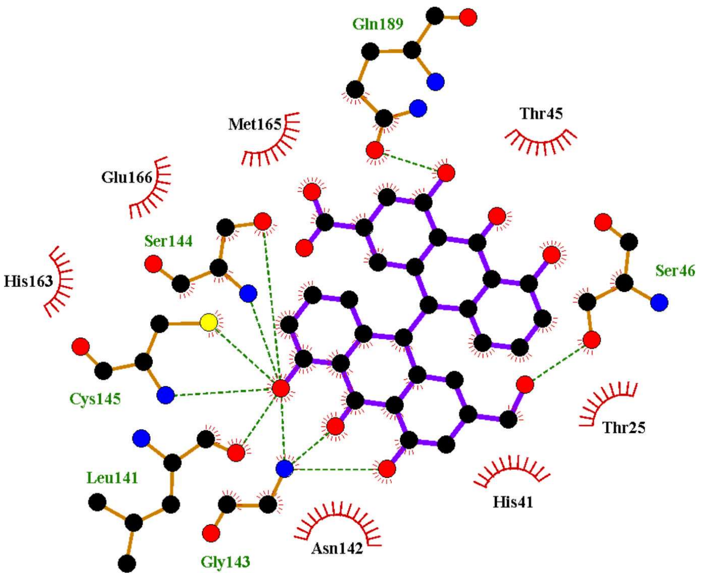

ZINC000100826931

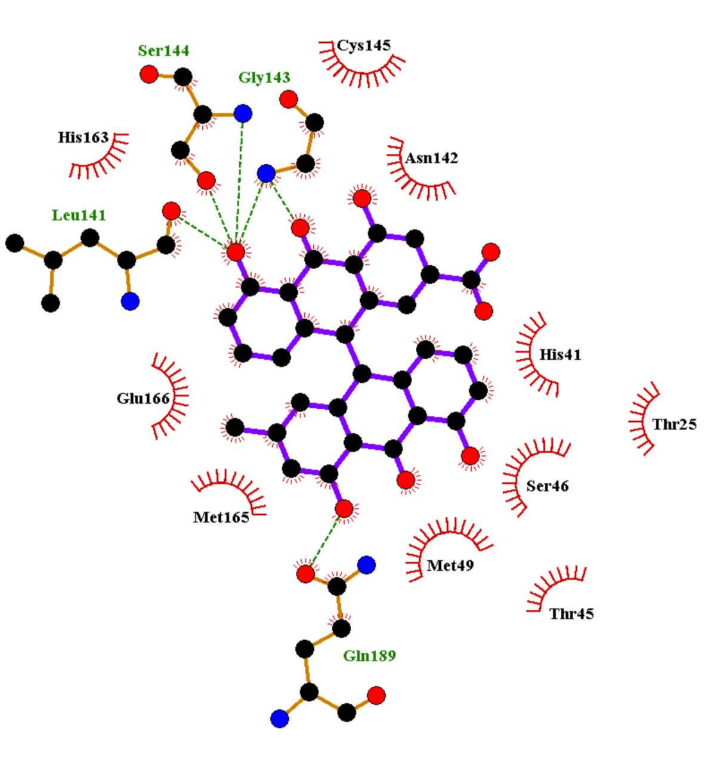

ZINC000072267001

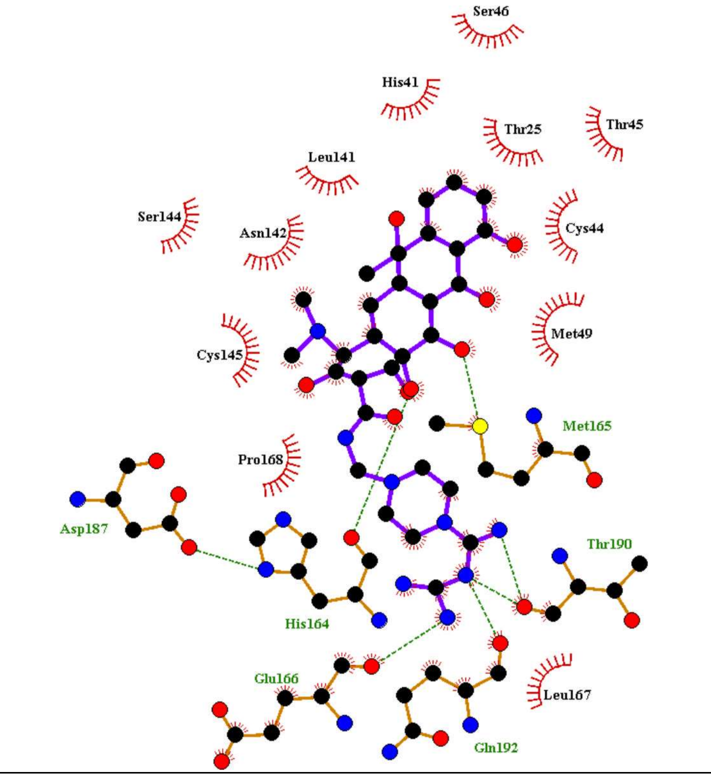

ZINC000100777664

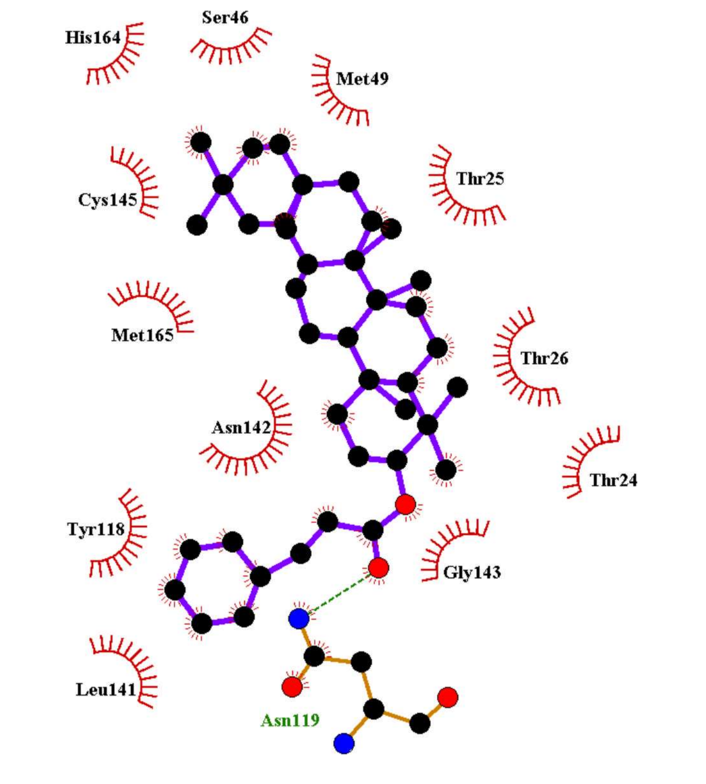

ZINC000085974153

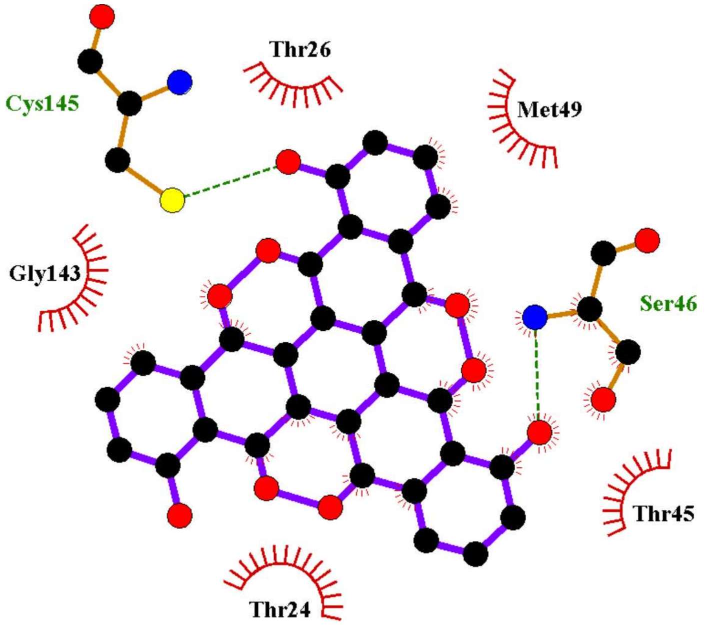

Table S3. The obtained values of the molecular docking and FPL simulations.

N⁰	ZINC ID	Name	ΔG_{Dock} [a]	F_{Max} [b]	W [c]	ΔG_{FPL}^{Pre} [d]
1	ZINC000253599724	Asparagoside A	-8.9	534.1 ± 41.0	61.9 ± 3.8	-8.96
2	ZINC000100774275	Rubroskyrin	-9.0	579.1 ± 30.2	60.9 ± 3.5	-8.91
3	ZINC000004215844	Etamestrol	-9.0	560.6 ± 39.0	59.5 ± 4.3	-8.83
4	ZINC000085537129	Penimocycline	-8.9	569.8 ± 25.6	58.0 ± 3.1	-8.75
5	ZINC000100774279	Deoxyrubroskyrin	-9.2	582.1 ± 32.9	57.7 ± 5.6	-8.73
6	ZINC000100777465	Physalin G	-9.2	637.5 ± 43.4	57.6 ± 4.2	-8.72
7	ZINC000113198271	Altiratinib	-8.9	533.4 ± 25.1	57.2 ± 3.3	-8.70
8	ZINC000085488845	Bisisodiospyrin	-9.6	559.1 ± 38.9	56.9 ± 4.2	-8.69
9	ZINC000095564635	Afoxolaner	-8.9	485.8 ± 29.1	56.8 ± 4.0	-8.68
10	ZINC000100772783	Asticolorin A	-9.3	596.3 ± 33.8	56.7 ± 2.5	-8.68
11	ZINC000100826921	Rheidin A	-9.8	608.3 ± 24.5	55.1 ± 1.9	-8.59
12	ZINC000051951668	R428	-9.0	550.1 ± 28.3	54.9 ± 2.4	-8.57
13	ZINC000100822646	Palmidin C	-9.9	604.0 ± 24.7	54.0 ± 2.5	-8.53
14	ZINC000004214742	Undefined	-9.1	512.6 ± 23.5	53.9 ± 3.2	-8.52
15	ZINC000239329011	Limonin	-9.1	610.3 ± 41.0	53.3 ± 2.5	-8.49
16	ZINC000118937393	2alpha-Hydroxypyracrenic acid	-9.0	446.6 ± 31.0	53.3 ± 5.3	-8.48
17	ZINC000103941727	Physalin B	-9.0	578.8 ± 19.5	53.1 ± 1.8	-8.47
18	ZINC000043061730	Flocoumafen	-9.0	550.8 ± 14.7	53.0 ± 2.4	-8.47
19	ZINC000256421903	Asperagenin	-9.0	589.6 ± 43.0	52.2 ± 3.0	-8.42
20	ZINC000150353464	7-Epizucchini Factor A	-9.1	561.3 ± 43.5	52 ± 4.2	-8.41
21	ZINC000253527861	Glycyrrhizin	-8.9	513.4 ± 47.2	52 ± 6.2	-8.41
22	ZINC000100772795	Asticolorin B	-8.9	570.4 ± 37.7	51.6 ± 3.3	-8.39
23	ZINC000095619883	Jangomolide	-9.2	590.7 ± 30.5	51.2 ± 2.7	-8.37
24	ZINC000100822774	Palmidin B	-9.2	567.3 ± 30.7	50.9 ± 2.5	-8.35
25	ZINC000100783375	25,27-Dihydro-4,7-didehydro-7-deoxyphysalin A	-9.1	571.5 ± 18.1	50.5 ± 1.7	-8.33
26	ZINC000100783373	4,7-Didehydroneophysalin B	-9.7	591.2 ± 54.3	50.4 ± 6.8	-8.32

27	ZINC000261496785	Chaetoglobosin N	-9.2	549 ± 29.9	49.6 ± 2.1	-8.28
28	ZINC000100826926	Rheidin C	-9.9	568.6 ± 42.4	48.8 ± 4.1	-8.23
29	ZINC000100777456	Physalin E	-9.2	487.4 ± 42.0	46.4 ± 3.8	-8.1
30	ZINC000100779057	Corosin	-8.9	502.3 ± 32.9	45.5 ± 2.7	-8.05
31	ZINC000118937472	3-O-p-trans-Coumaroylalphitolic acid	-9.0	449.3 ± 22.7	45.5 ± 2.8	-8.05
32	ZINC000100783384	Isophysalin B	-9.0	509.3 ± 24.4	44.5 ± 2.8	-7.99
33	ZINC000100777392	Physalin H	-9.2	467.4 ± 14.5	44.2 ± 1.9	-7.98
34	ZINC000100782938	Physalin N	-9.4	499.6 ± 38.2	43.9 ± 3.9	-7.96
35	ZINC000150353094	Zucchini Factor B	-9.3	411.8 ± 18.2	42.2 ± 1.7	-7.87
36	ZINC000100783281	Physalin P	-9.1	482.8 ± 19.4	38.9 ± 2.5	-7.84
37	ZINC000070450821	Sennidin C	-9.1	406.0 ± 18.0	38.1 ± 3.1	-7.64
38	ZINC000100826931	Rheidin B	-9.7	450.9 ± 37.5	37.2 ± 4.4	-7.58
39	ZINC000072267001	Guamecycline	-9.4	409.8 ± 41.9	36.7 ± 6.1	-7.56
40	ZINC000100777664	Germanicol Cinnamate	-9.6	389.5 ± 20.9	34.5 ± 2.2	-7.44
41	ZINC000085974153	Trisjuglone	-9.3	406.2 ± 16.0	32.9 ± 2.6	-7.35

[a]The docking affinity was calculated using the Autodock Vina package. [b]The obtained value of the mean rupture force $\boldsymbol{F}_{\mathbf{Max}}$ and [c]the recorded metric of the pulling work $\boldsymbol{W}$. [d]The predicted binding free energy $\Delta G_{\mathrm{FPL}}^{\mathrm{Pre}}$ was attained using Eq. (1). The computed error was the standard error of the average. The unit of energy and force are in kcal mol^{-1} and pN, respectively.

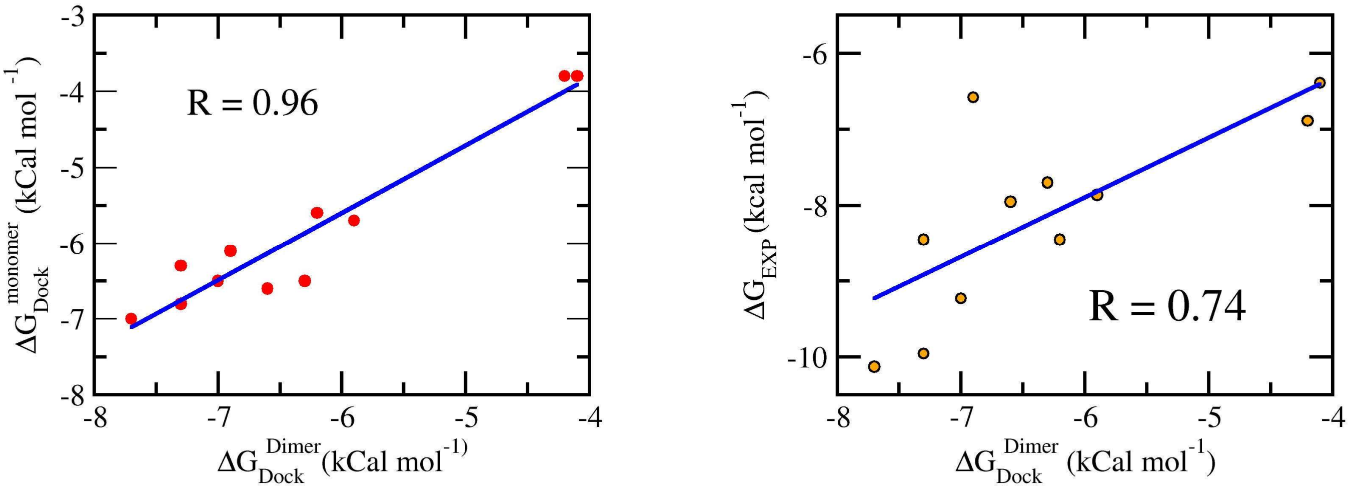

Figure S1. Association between the docking energy $\Delta G_{\mathrm{Dock}}^{\mathrm{monomer}}$ versus $\Delta G_{\mathrm{Dock}}^{\mathrm{Dimer}}$ (left) and $\Delta G_{\mathrm{Dock}}^{\mathrm{Dimer}}$ versus ΔG_{EXP} (right).

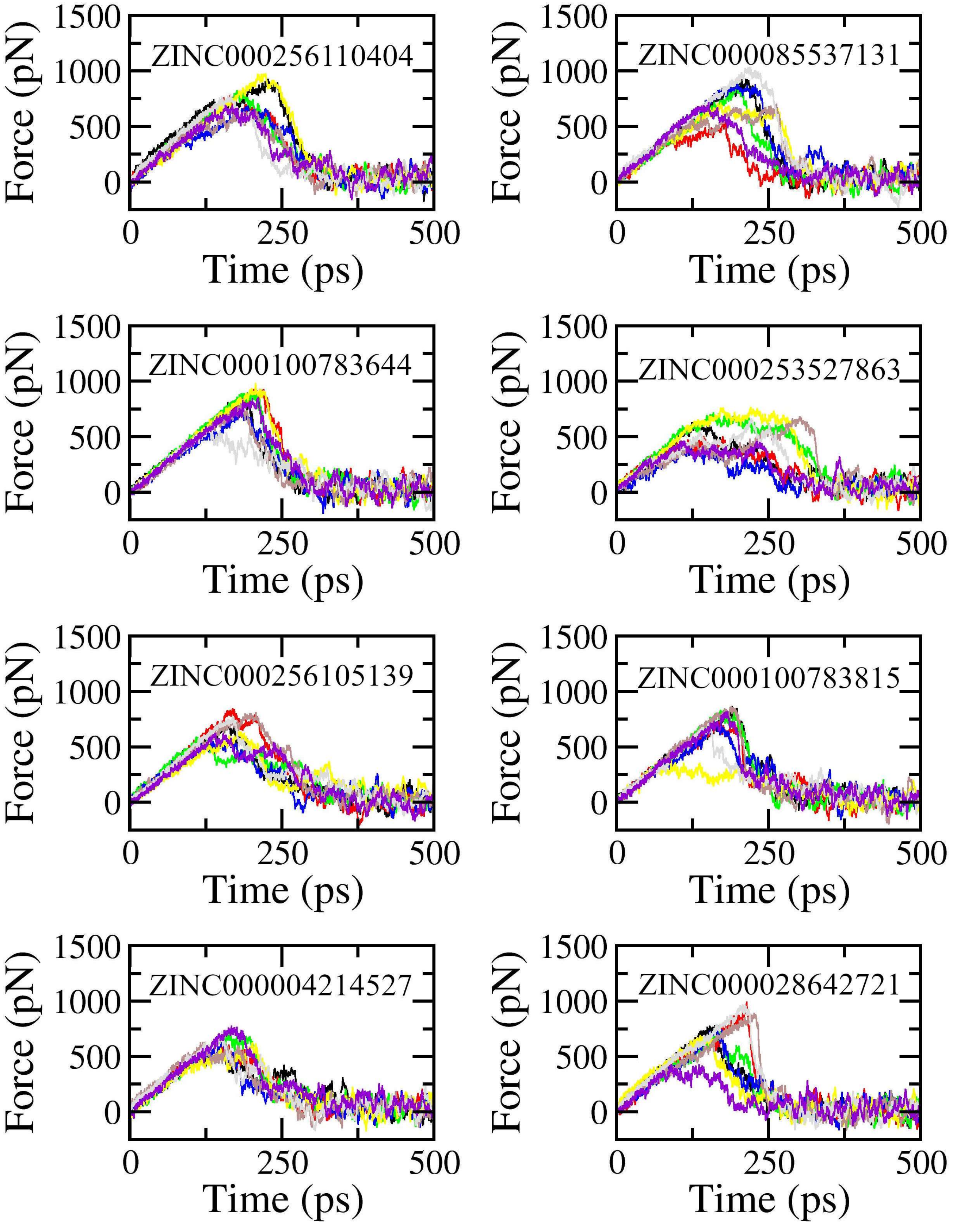
ZINC000256110404
ZINC000085537131
ZINC000100783644
ZINC000253527863
ZINC000256105139
ZINC000100783815
ZINC000004214527
ZINC000028642721
Force (pN)
Time (ps)
0
250
500
1500
1000
500
0

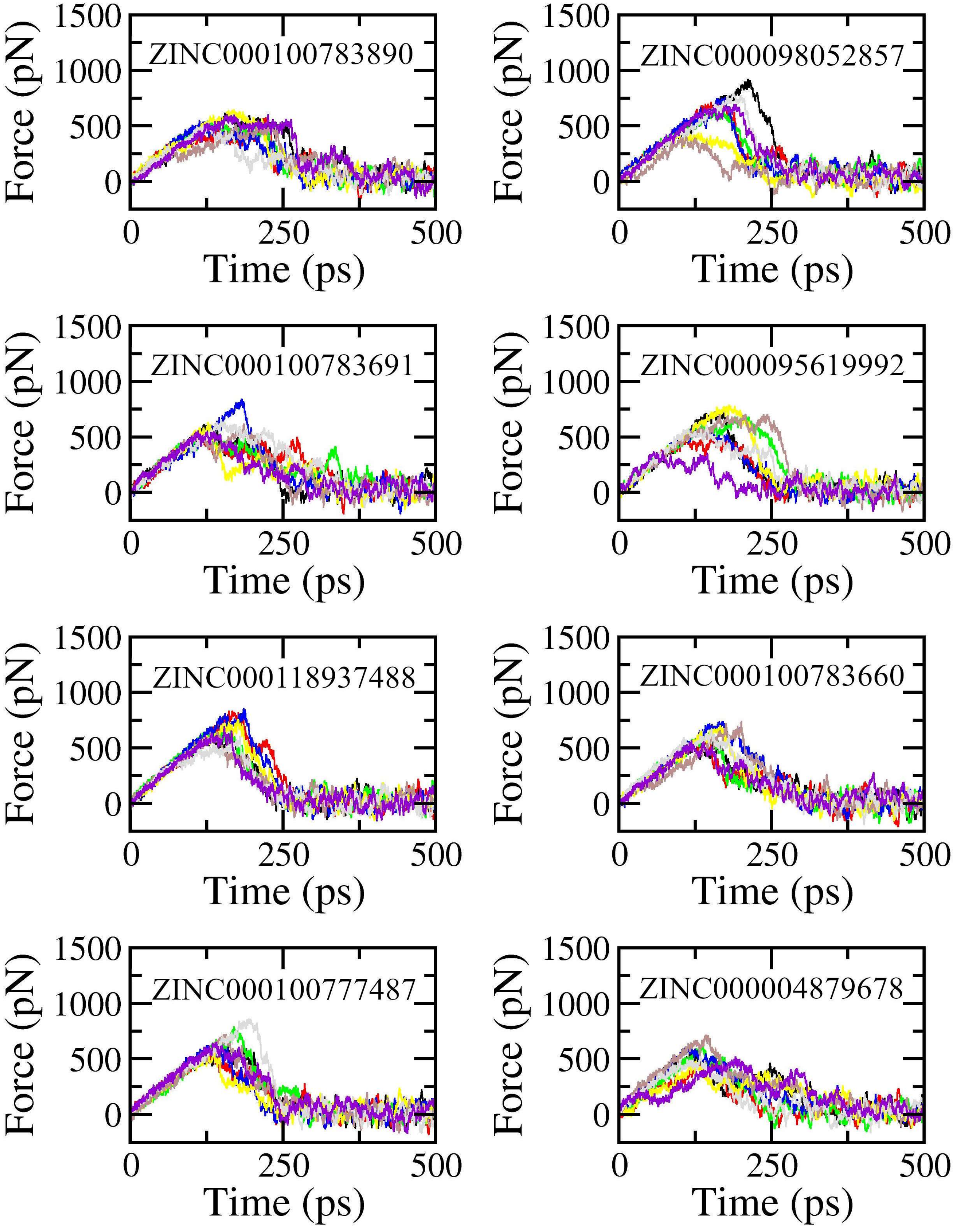
ZINC000100783890
ZINC000098052857
ZINC000100783691
ZINC000095619992
ZINC000118937488
ZINC000100783660
ZINC000100777487
ZINC000004879678
Force (pN)
Time (ps)
0
250
500
1500
1000
500
0

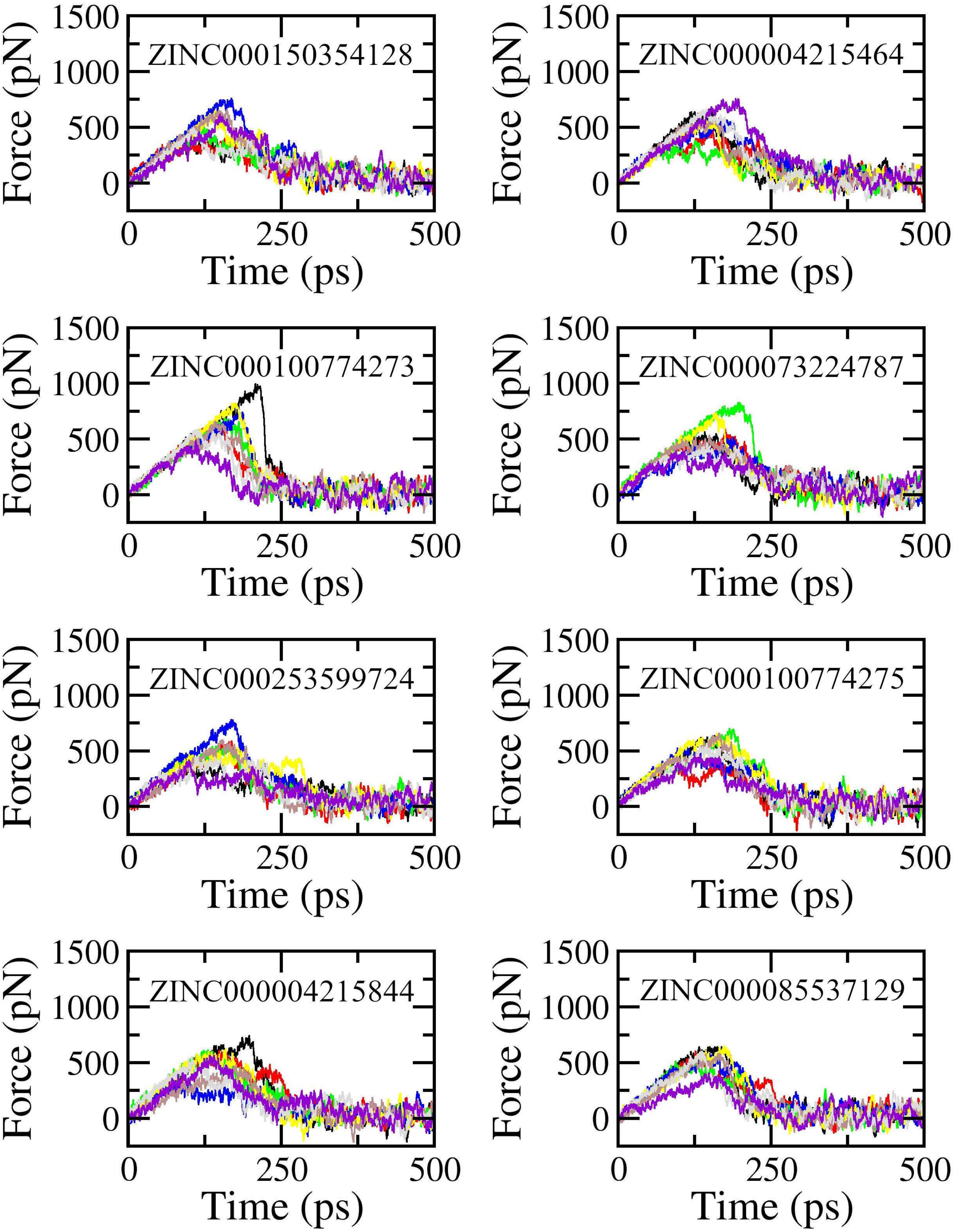
ZINC000150354128
ZINC000004215464
ZINC000100774273
ZINC000073224787
ZINC000253599724
ZINC000100774275
ZINC000004215844
ZINC000085537129
Force (pN)
1500
1000
500
0
Time (ps)
0
250
500

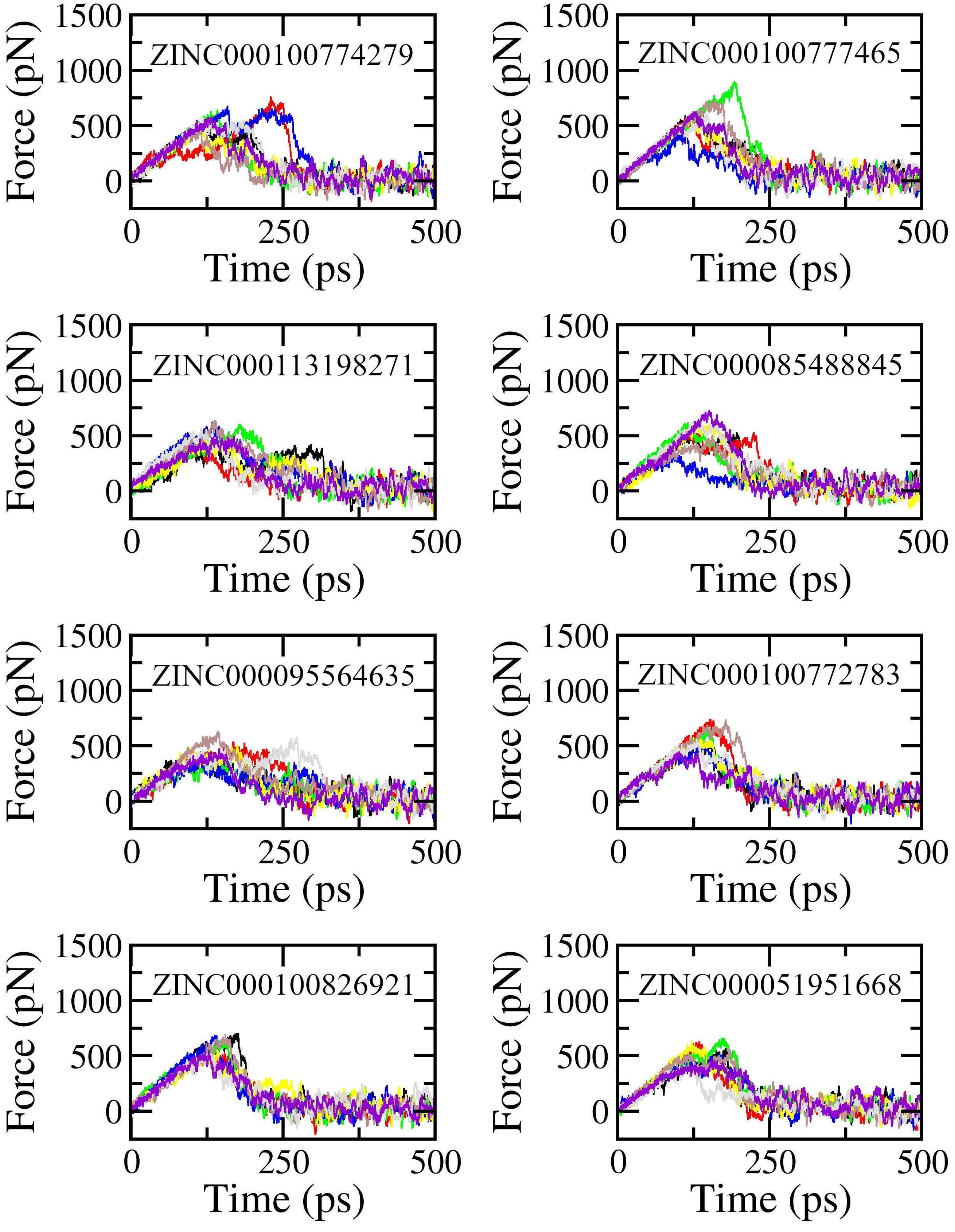
ZINC000100774279
ZINC000100777465
ZINC000113198271
ZINC000085488845
ZINC000095564635
ZINC000100772783
ZINC000100826921
ZINC000051951668
Force (pN)
0
500
1000
1500
Time (ps)
0
250
500

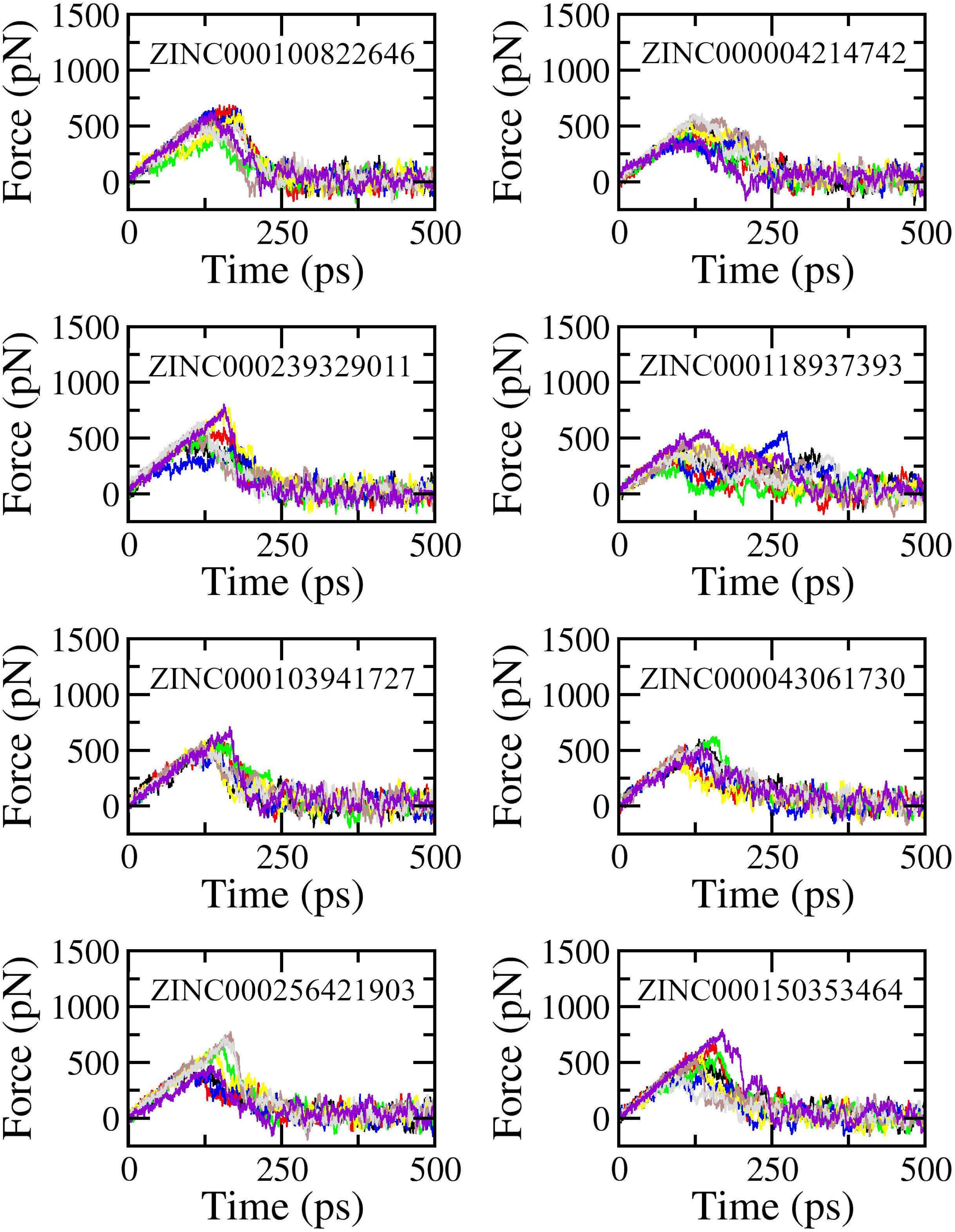
ZINC000100822646
ZINC000004214742
ZINC000239329011
ZINC000118937393
ZINC000103941727
ZINC000043061730
ZINC000256421903
ZINC000150353464
Force (pN)
Time (ps)
1500
1000
500
0
250

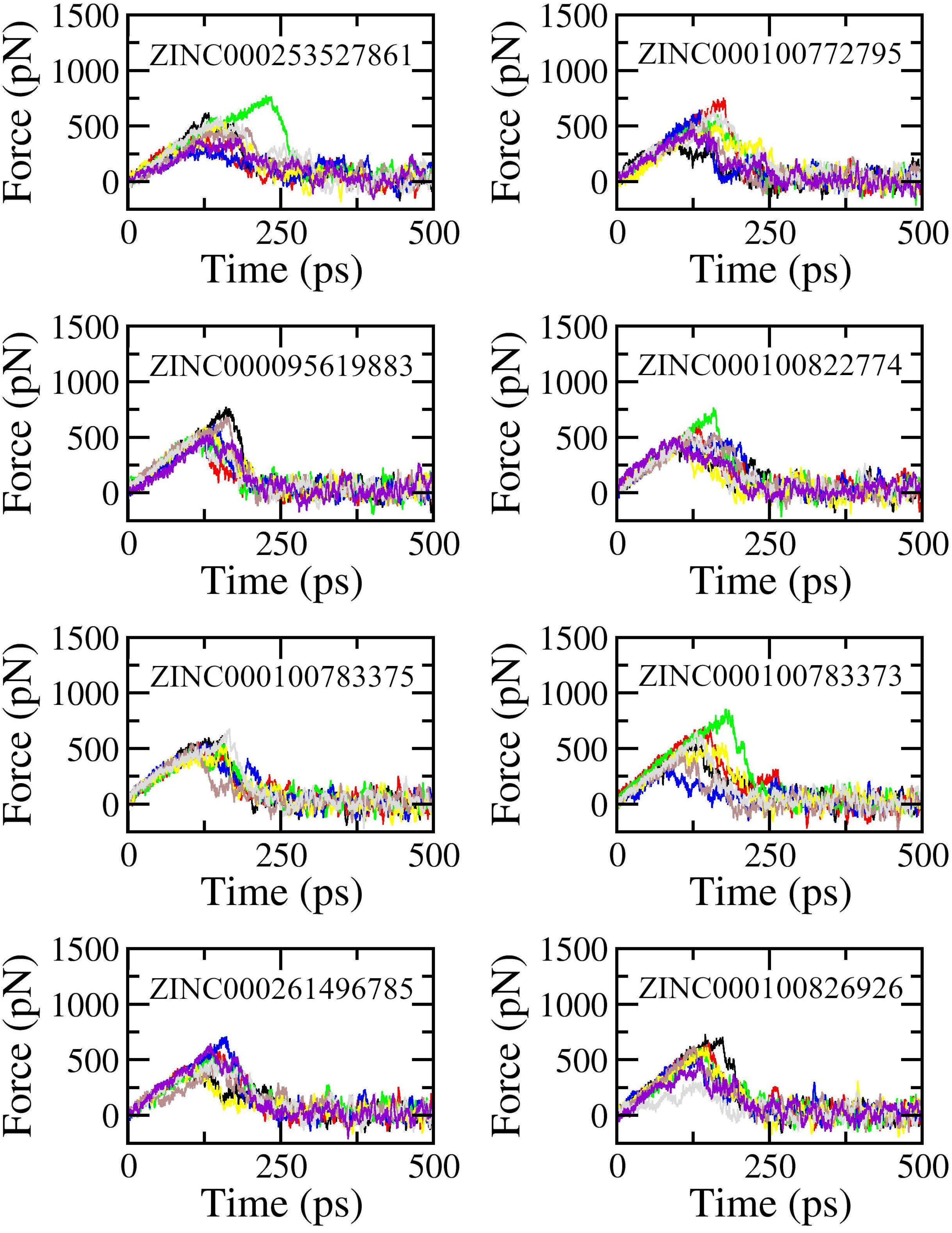
ZINC000253527861
ZINC000100772795
ZINC000095619883
ZINC000100822774
ZINC000100783375
ZINC000100783373
ZINC000261496785
ZINC000100826926
Force (pN)
Time (ps)
0
250
500
1000
1500

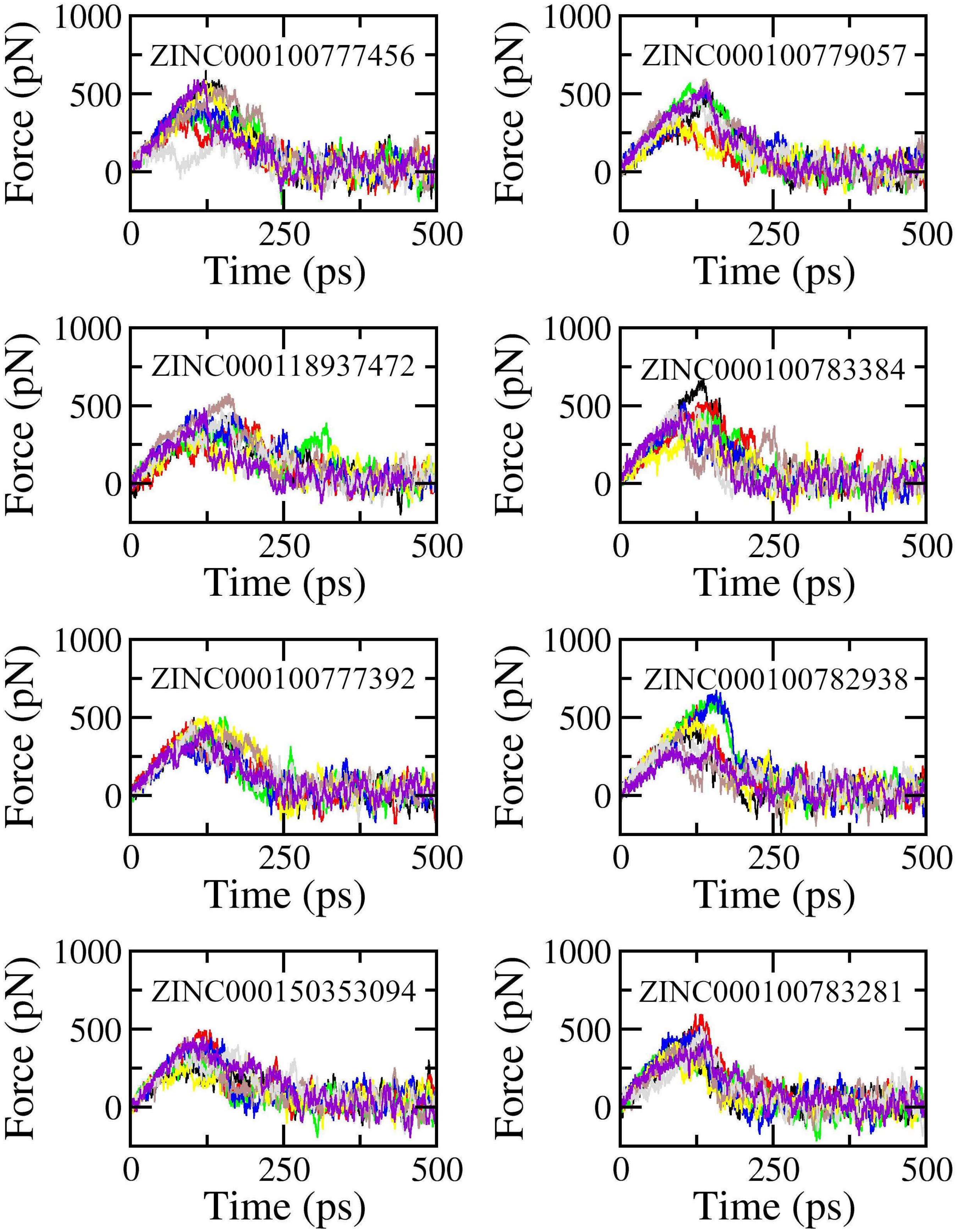
ZINC000100777456
ZINC000100779057
ZINC000118937472
ZINC000100783384
ZINC000100777392
ZINC000100782938
ZINC000150353094
ZINC000100783281
Force (pN)
Time (ps)
1000
500
0
250
500

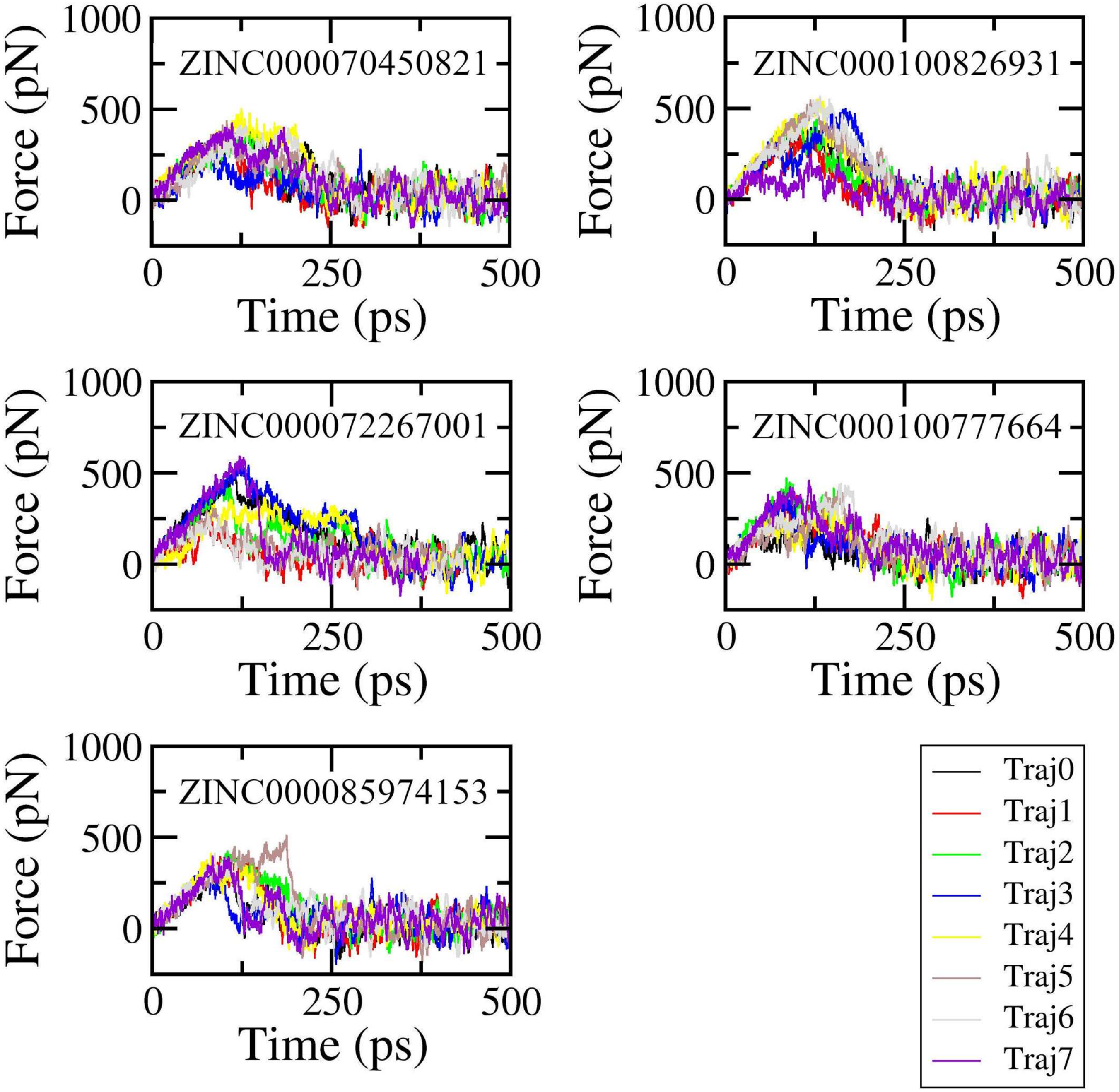

Figure S2. Externally pulling forces in time dependence over 8 independent FPL trajectories.
